# A fact based analysis of decision trees for improving reliability in cloud computing

**DOI:** 10.1371/journal.pone.0311089

**Published:** 2024-12-03

**Authors:** Muhammad Asim Shahid, Muhammad Mansoor Alam, Mazliham Mohd Su’ud

**Affiliations:** 1 Malaysian Institute of Information Technology, Universiti Kuala Lumpur, Kuala Lumpur, Malaysia; 2 School of Computing and Information Sciences, Sohail University, Karachi, Pakistan; 3 Faculty of Computing, Riphah International University, Islamabad, Pakistan; 4 School of Computer Science, University of Technology Sydney, Ultimo, NSW, Australia; 5 Persiaran Multimedia, Multimedia University, Cyberjaya, Malaysia; Najran University College of Computer Science and Information Systems, SAUDI ARABIA

## Abstract

The popularity of cloud computing (CC) has increased significantly in recent years due to its cost-effectiveness and simplified resource allocation. Owing to the exponential rise of cloud computing in the past decade, many corporations and businesses have moved to the cloud to ensure accessibility, scalability, and transparency. The proposed research involves comparing the accuracy and fault prediction of five machine learning algorithms: AdaBoostM1, Bagging, Decision Tree (J48), Deep Learning (Dl4jMLP), and Naive Bayes Tree (NB Tree). The results from secondary data analysis indicate that the Central Processing Unit CPU-Mem Multi classifier has the highest accuracy percentage and the least amount of fault prediction. This holds for the Decision Tree (J48) classifier with an accuracy rate of 89.71% for 80/20, 90.28% for 70/30, and 92.82% for 10-fold cross-validation. Additionally, the Hard Disk Drive HDD-Mono classifier has an accuracy rate of 90.35% for 80/20, 92.35% for 70/30, and 90.49% for 10-fold cross-validation. The AdaBoostM1 classifier was found to have the highest accuracy percentage and the least amount of fault prediction for the HDD Multi classifier with an accuracy rate of 93.63% for 80/20, 90.09% for 70/30, and 88.92% for 10-fold cross-validation. Finally, the CPU-Mem Mono classifier has an accuracy rate of 77.87% for 80/20, 77.01% for 70/30, and 77.06% for 10-fold cross-validation. Based on the primary data results, the Naive Bayes Tree (NB Tree) classifier is found to have the highest accuracy rate with less fault prediction of 97.05% for 80/20, 96.09% for 70/30, and 96.78% for 10 folds cross-validation. However, the algorithm complexity is not good, taking 1.01 seconds. On the other hand, the Decision Tree (J48) has the second-highest accuracy rate of 96.78%, 95.95%, and 96.78% for 80/20, 70/30, and 10-fold cross-validation, respectively. J48 also has less fault prediction but with a good algorithm complexity of 0.11 seconds. The difference in accuracy and less fault prediction between NB Tree and J48 is only 0.9%, but the difference in time complexity is 9 seconds. Based on the results, we have decided to make modifications to the Decision Tree (J48) algorithm. This method has been proposed as it offers the highest accuracy and less fault prediction errors, with 97.05% accuracy for the 80/20 split, 96.42% for the 70/30 split, and 97.07% for the 10-fold cross-validation.

## Introduction

### Research background & motivation

Over the last decade, many corporations & business sectors have shifted to cloud computing to ensure transparency, scalability, & accessibility due to its exponential growth. Due to cost savings & complex resource distribution, many people switch to the cloud where the infrastructure is managed by cloud providers [1]. Cloud service providers such as International Business Machines (IBM), Amazon, Yahoo, & Google offer cloud computing services to consumers worldwide [2]. Cloud architectures refer to the transfer of computational services amongst multiple users. In such architectures, apps, hardware, & software systems are shared resources. Typically, cloud architectures are composed of three main layers: Infrastructure as a Service (IaaS), Software as a Service (SaaS), & Platform as a Service (PaaS). Cloud infrastructure deficiencies can significantly affect the reliability of resources. To enhance the reliability & effectiveness of cloud computing, it is important to identify & fix any defects that may arise [3]. Users can access cloud computing resources over the internet & pay for them based on usage. The cloud provider responsible for cloud processing outsources every resource that belongs to the customer [4]. Smart gadgets have proven to be beneficial due to their improved functionality, offering tools that are always available to suit a user’s needs, no matter where they are [5].

The Antarex secondary dataset is a collection of trace data gathered during fault injection experiments on the Eidgenössische Technische Hochschule Zurich (ETH) experimental High Performance Computing (HPC) system. Its purpose is to enable Machine Learning (ML) based HPC system fault detection research. The dataset is split into two sections: one for benchmark applications & fault programs related to CPU & Random Access Memory (RAM), & another for applications & fault programs linked to hard drives. There are four folders in the Antarex dataset, one corresponding to each dataset block (CPU/Memory & HDD), & both in single-core & multi-core versions [6]. The Weibull distribution technique is used to create the fundamental dataset. In the field of dependability, the Weibull distribution is commonly used as a model for time-to-failure. It allows for non-constant failure rate functions, which expands the capabilities of the exponential model. This model can be used to describe both early burning & wear-out failures, & it includes both increasing & decreasing failure rate curves [7].

Thanks to the growth of machine learning (ML) & increased accessibility of building data, there is immense potential to apply ML to model & analyze building energy systems. Situated at the intersection of computer science & statistics, ML is a rapidly growing data-driven field that plays a crucial role in artificial intelligence (AI) & data science [8]. Computers can learn from supplied data & ML techniques, without requiring explicit programming for each problem. By identifying deep connections within data inputs, it tries to reconstruct a knowledge structure. The learning outcomes can be utilized for categorization, prediction, & estimation purposes [9]. There are two main types of ML techniques: supervised & unsupervised. In supervised learning, an AI network is trained to create a mapping function that maps input data to output using a dataset of input & target values. Unsupervised learning is a type of ML that involves the use of an input dataset that is neither labeled nor classified. The AI network is trained to discover hidden patterns, solutions, & distributions without any guidance. Unsupervised learning challenges come in several forms, such as association & clustering [10].

### Research gaps

Identifying research gaps in previous studies is essential to broaden the article’s impact. After reviewing the relevant body of knowledge, the literature evaluation uncovered the following research gaps.

- The decision tree model does not consider reliability [10].
- One of the most critical research gaps faced by scholars and practitioners in the cloud computing environment within decision tree frameworks is reliability [10].
- Based on the literature review, researchers should develop an algorithm using machine learning methods to enhance the reliability of cloud nodes [11].
- The machine learning of cloud systems has garnered significant attention for its creative applications. Few studies have explored using machine learning to enhance the reliability of cloud systems [11].
- A new approach is required for machine learning in cloud environments to provide maximum stability across virtual machines, minimum failure prediction, and high accuracy [12].

### Research objectives

This research aims to improve the quality of service (QoS) in CC by using ML to reduce fault prediction errors & increase accuracy. To achieve this goal, the following objectives must be met:

- Find out how decision trees can enhance the reliability measures of cloud computing systems.
- Determine the main factors that influence the reliability of cloud computing systems and assess the accuracy of decision trees in representing and predicting these factors.
- Compare and contrast multiple machine learning algorithms (e.g. Deep Learning, Bagging, Decision Tree, AdaBoost M1, and Naive Bayes Tree) to determine the most effective in predicting and addressing reliability issues in cloud systems.

### Research scope

The following research scope has been strictly adhered to:

#### Data gathering

The secondary dataset consists of trace data from the same experimental HPC system at ETH, Zurich, while the primary dataset provides repair and failure Virtual Machines (VMs) data to support an ML-based strategy for Fault Tolerance (FT) dependability in cloud computing.

#### Machine learning algorithms

Supervised machine learning techniques are utilized in this study to enhance accuracy and reduce fault prediction errors. It involves the use of labeled datasets to train algorithms for proper data identification and outcome prediction.

### Reliability

Reliability in the context of cloud computing refers to a system’s ability to perform its intended function or provide the required service for a specific duration under predefined conditions. In this study, we achieved reliability through the use of machine learning. The ability of each virtual machine to operate continuously is what we define as reliability.

### Contribution of the study

We have made significant progress in our efforts. Firstly, we acquired the HPC fault dataset & tested a fault classification technique based on supervised ML. We are pleased to share that the dataset & all test environment details are publicly available for the community to use. Additionally, we will be utilizing trace data from the Antarex experimental HPC system at ETH Zurich to create a secondary dataset for ML-based failure prediction studies. The Antarex dataset will be divided into two parts: benchmark apps & fault programs connected to CPU & RAM, & applications & fault programs linked to hard drives. To make it more organized, we have categorized the dataset into four folders, one for each dataset block (CPU/Memory & HDD) in both single-core & multi-core versions. This will help researchers & academics in their studies & experiments [11].

We utilized the Weibull distribution technique to create a primary dataset. The Weibull distribution is another commonly used model to predict the time-to-failure in reliability. Unlike the exponential model, it incorporates failure rate functions that are not constant, thus providing a more comprehensive understanding of wear-out failures, early burnings, & both rising & declining failure rate curves [38]. Various Java platform settings were programmed to generate primary data using the Weibull distribution technique.

We utilized two types of datasets for our study: Antarex Secondary Datasets & Primary Datasets. The secondary dataset was obtained from the ZONODO website & the primary data was produced using the Weibull distribution technique [11, 12]. We conducted tests to identify the most effective ML algorithms in terms of high accuracy & less fault prediction, & we present our findings in this regard.

The J48 decision tree classifier has proven to be highly accurate & effective in reducing fault prediction errors, making it a valuable tool for CC users. This marks the fourth & final contribution.

## Materials and methods

### Literature review

Shahid et al. [2] suggested that CC has become a prominent trend in recent times. This has led to the development of large-scale computer networks from previously dispersed systems. Globally, companies such as IBM, Amazon, Yahoo, & Google provide cloud services to their clients. This new paradigm enables software & services to be provided to end-users on demand, doing away with the need for them to install programs on their local computers.

Shahid et al. [3] investigate that cloud architectures are designed to enable the exchange of computing resources among different users. These shared resources include software, hardware, & applications. IaaS, SaaS, & PaaS are the three primary layers that make up most cloud infrastructures. Although errors can occur at any of these levels, recovery techniques are identified & applied at the software level to ensure smooth operation.

Mishra et al. [13] researchers have developed various load-balancing techniques to optimize different performance metrics in CC. They have categorized these cloud-based load-balancing algorithms & explored their impact on the literature. To examine the performance of heuristic-based algorithms, a simulation was run using the CloudSim simulator. The outcomes of the simulation have been provided in detail.

Feng et al. [14] this research presents a smart approach to predict the compressive strength of concrete using ML technology. The method combines multiple weaker learners through an adaptive boosting technique to create a robust learner that can effectively establish the correlation between the input & output data.

Butt et al. [15] in this review paper, an analysis of security threats, issues, & solutions related to CC that utilize one or several ML algorithms is presented. They discuss various ML algorithms that are used to tackle cloud security issues, including supervised, unsupervised, semi-supervised, & reinforcement learning. Then, we compare the performance of each strategy based on their features, advantages, & downsides. Additionally, we highlight possible research directions to ensure the security of CC models.

Pei et al. [16] a new fault prediction model called multidimensional fusion (CNN-BiLSTAM-Attention) (CBA)-net has been proposed. The model is based on Hierarchical Density-Based Spatial Clustering of Applications with Nois (HDBSCAN) clustering preprocessing classification data & can effectively extract & learn spatial & temporal features from the predecessor fault log. It can extract local features & is highly sensitive to time series features. The experiments conducted on the model demonstrated that it has an Root Mean Square Error (RMSE) of 0.031 for fault occurrence time prediction & an average prediction accuracy of 93% for node location during fault occurrence. The model can improve the fine-grained & accurate fault prediction of large supercomputers by achieving fast convergence.

Shrestha & Mahmood [17] in this study, various optimization techniques are examined, which can be used to reduce the duration of training while simultaneously enhancing the accuracy of training. The research delves into the mathematical principles behind the training techniques that are commonly used in modern deep networks. The paper outlines the current shortcomings, improvements, & practical applications of these techniques. In addition, the paper covers a range of deep architectures, such as variation autoencoders, recurrent neural networks, deep residual networks, reinforcement learning, & deep convolution networks.

Lang et al. [18] suggested that Deep learning is a specific area of ML that employs artificial neural networks to produce multi-layered data representations. This technology has significantly improved the performance of various ML tasks, such as document classification, object detection, speech recognition, & image classification. Introducing WekaDeeplearning4j - a Weka module that provides a graphical user interface (GUI) for easy access to deep learning. This software allows for the GUI-based training of deep neural networks, including convolutional & recurrent neural networks, & it utilizes Deeplearning4j as its backend. It also supports Graphics Processing Unit (GPUs) & includes pre-processing tools for text & image data.

Wang et al. [19] proposed a novel approach known as the multinomial naive Bayes tree (MNBTree) has been proposed by implementing a multinomial naive Bayes text classifier on each leaf node of the decision tree. Unlike NBTree, MNBTree creates a binary tree where the split attributes are divided into zero & nonzero values. MNBTree builds the tree faster by using the information gain metric instead of the classification accuracy measure. To further improve the classification performance of MNBTree, the multiclass multinomial naive Bayes tree (MMNBTree) is suggested, which uses the multiclass approach. The experimental findings on various popular text categorization benchmark datasets have validated the effectiveness of our proposed methods, MNBTree & MMNBTree.

Bildosola et al. [20] this study outlines a practical & proven tool that can be used as a decision-making resource for adopting CC. The tool involves a diagnosis process based on predetermined questions to collect the necessary data & provide the user with useful information to launch their business in the cloud, specifically through the use of Software as a Service (SaaS) solutions. With this information, decision-makers can create their own customized Cloud Roadmap. The pilot research involved local businesses & had two objectives: firstly, to determine the level of knowledge people had about CC; & secondly, to identify the most promising industries & the tools that are best suited for them.

Jaiganesh et al. [21] suggested using a priority-based queuing model to evaluate the leased services provided by cloud service providers. This model takes into account the overall service time & reaction time for incoming requests & uses a queue to hold waiting requests. The services offered by providers are classified as Platform as a Service (PaaS), Infrastructure as a Service (IaaS), & Software as a Service (SaaS). The queuing model we build includes a buffer of size ’r’, a priority queue discipline, a Markovian arrival rate, a general service rate, & ’m number of servers. The benefit of using this analytical model is that the cloud service provider can arrange their services to maximize profit within a given time frame.

Batista et al. [22] this study evaluated the performance of a cloud resource management module. The module manages available resources during execution time & ensures the service quality specified in the service level agreement. To determine which aspect of resource scaling affects client requests, various resource configurations were analyzed. The study’s outcomes were used to create a model & implement a simulated cloud system. The allotted resource can be modified at any time & a different cost. The suggested module aims to satisfy both the supplier & the customer by guaranteeing the highest level of service quality & the most economical use of resources.

Qiu et al. [23] present a survey of the latest research advances in ML for big data processing. Firstly, the paper reviews various ML techniques that have been developed & discusses some promising learning methods from recent studies like representation, deep, distributed, parallel, transfer, active, & kernel-based learning. Next, the paper delves into the challenges & potential solutions of ML for big data, & provides a detailed analysis of the same.

Zhang et al. [24] this study explores different strategies for weighting features in text classifiers that use the naive Bayes algorithm. Most existing feature weighting methods for such classifiers have some drawbacks, such as making the models more complex & slower, or only providing marginal improvements in classification performance. However, the ML community has a long history of research into feature weighting, & many scholars have made important contributions to this field. In addition, discusses a few straightforward & effective feature weighting techniques that were created for naive Bayes classifiers in general & modified for use with naive Bayes text classifiers.

Liu et al. [25] suggested that failure detectors are an important part of high-availability distributed systems. Accrual failure detectors, in particular, have been extensively studied to meet the needs of complex, multi-application distributed systems. However, some implementations of accrual failure detectors face challenges in adapting to the context of cloud services. A new accrual failure detector called the Weibull Distribution Failure Detector has been designed specifically for CC. This solution is based on the Weibull Distribution & can adapt to the unpredictable & changing network conditions often seen in CC. The performance of the Weibull Distribution Failure Detector has been evaluated & compared to data from both CC & public classical experiments. The results show that the Weibull Distribution Failure Detector is faster & more accurate in unstable conditions, particularly in CC.

Navimipour & Vakili [26] suggested that CC typically involves the deployment of virtualized resources that are dynamically scalable as services over the Internet. Depending on the user’s needs, various services can be provided in the cloud environment, which may need to be combined to meet the user’s expectations. As a result, service composition has become a widely used technique for integrating different services in the cloud environment to aggregate & consolidate them. This approach focuses on creating a new cloud service that combines existing services to reduce costs, save time, & increase efficiency. This paper aims to provide an overview of the methods & approaches currently utilized in the field of cloud service composition. In summary, this paper makes three contributions: Firstly, it offers a general overview of the challenges that exist in various problem domains related to cloud service composition. Secondly, it describes some key techniques used within the scope of cloud service composition. And finally, it identifies important areas for future research to enhance service composition methods.

Madni et al. [27] investigated that CC infrastructure is suitable for managing large processing tasks. However, scheduling jobs in CC environments presents an NP-complete problem that requires heuristic solutions. A variety of heuristic algorithms have been developed & used to address this issue. However, selecting the most appropriate algorithm to solve a particular job assignment problem can be challenging because the approaches were developed based on different assumptions. Six rule-based heuristic algorithms have been developed to schedule autonomous activities in both homogeneous & heterogeneous contexts. These algorithms are used to compare the performance of task scheduling in CC in terms of cost, degree of imbalance, makespan, & throughput. The heuristic methods considered for this analysis include First Come First Serve (FCFS), Minimum Completion Time (MCT), Minimum Execution Time (MET), Maxmin, Min-min, & Sufferage.

Tanha et al. [28] suggested that in ML, some methods consider both labeled & unlabeled data for learning tasks. One such method is semi-supervised learning, which involves self-training using decision tree learners as the base learners. However, we have demonstrated that ordinary decision tree learning cannot be used as a basic learner for self-training in semi-supervised learning. The primary reason for this is that the fundamental decision tree learner is unable to provide accurate probability estimates for its predictions. The researchers considered various techniques such as Naive Bayes Tree, grafting, distance-based metric, & a combination of no-pruning & Laplace correction to improve decision tree algorithms. They also extended this enhancement to decision tree ensembles & showed that the ensemble learner performs better than the modified decision tree learners, resulting in additional improvement.

Portugal et al. [29] this work aims to comprehensively evaluate the literature that examines the application of ML algorithms in recommender systems, to identify new research possibilities. The investigation has the following objectives: (i) recognize patterns in the application or investigation of ML algorithms in recommender systems; (ii) pinpoint unresolved issues in the application or investigation of ML algorithms; and (iii) support novice investigators in appropriately situating novel research endeavors within this field. The study’s findings describe the different types of recommender systems currently in use, the ML approaches that have been adopted, the use of big data technologies, & the identification of ML algorithm types & their application domains, & the analysis of both primary & secondary performance metrics.

Varghese & Buyya [30] the article discusses the evolution of cloud infrastructure & the benefits of shifting computing away from data centers. It also highlights the potential of new computer architectures that are expected to influence data-intensive computing, self-learning systems, linking people & things, & the service sector. The article concludes with a roadmap of obstacles that need to be addressed to fully utilize the potential of next-generation cloud systems.

Patel & Prajapati [31] suggested that ML is the process of teaching a computer to learn from different datasets, allowing it to determine its outcomes without explicit programming. One of the methods used in ML is Decision Trees. Decision Tree algorithms have a wide range of applications in various industries, such as search engines, medical fields, text extraction, & statistical analysis. Different decision tree algorithms have been developed based on their accuracy & effectiveness. It is crucial to know the optimal algorithm to use in different scenarios where a choice has to be made. This study focuses on three distinct decision tree algorithms: ID3, C4.5, & CART.

Mesbahi et al. [32] introduced WekaDeeplearning4j- a Weka package with a graphical user interface (GUI) that helps in deep learning. The software is capable of GUI-based training of deep neural networks, including convolutional & recurrent neural networks, & uses Deeplearning4j as its backend. It also supports GPUs & has pre-processing tools for text & picture data.

Table 1 presents a summary of the literature review from an important methodological perspective.

**Table 1:** An overview of the literature review.

Ref	Author Name	Year	Pros	Cons
[2]	Muhammad Asim Shahid et al.	2020	One of the main challenges in cloud systems is the need for efficient fault tolerance measures in algorithms, as emphasized in this study.	Their services are not reliable and of high quality.
[3]	Muhammad Asim Shahid et al.	2021	This review offers a comprehensive and detailed explanation of the different types of defects, variables, and fault tolerance techniques used in cloud computing.	Such traditional methods have disadvantages; they are often based on fixed principles and handle problems in a predetermined way, as indicated by their deployment.
[13]	Sambit Kumar et al.	2018	A detailed explanation is provided on how the system’s makespan and energy usage are calculated.	They have not tested the suggested algorithms in an actual cloud deployment.
[14]	De-Cheng Feng et al.	2019	Given the input variables, the AdaBoost model can effectively and accurately estimate the compressive strength of concrete.	The average MAPE is 11.39% and the average R2 of the 10-fold cross-validation is 0.952, indicating a poor prediction error.
[15]	Umer Ahmed Butt et al.	2020	In this review study, the researchers analyze cybersecurity threats, issues, and solutions involving one or more machine learning algorithms.	There is a need for a proposed approach to ensure dependability in the event of a virtual machine failure.
[16]	Xiangdong Pei et al.	2023	This study introduces a multi-dimensional fusion CBA-net (CNN-BiLSTAM-Attention) fault prediction model. It efficiently extracts and learns spatial and temporal characteristics from previous fault logs based on HDBSCAN clustering preprocessing classification data.	We will speed up pre-processing and data collection, improve the fault analysis and prediction mechanism and apply it to the system’s fault-tolerant recovery.
[17]	Ajay Shrestha and Ausif Mahmood	2019	This study explores various optimization techniques to reduce training times while improving accuracy.	Currently, overfitting, training duration, and the significant risk of becoming trapped in local minima limit training.
[18]	Steven Lang et al.	2019	They introduce WekaDeeplearning4j, a Weka package that provides a graphical user interface (GUI) for deep learning.	This extension does not enable the integration of deep learning models into users’ existing Weka workflows.

### Problem statement

Although decision trees are becoming more and more common in cloud computing, there is limited knowledge about their potential to improve system reliability. Currently, there is limited data available in the literature on the capacity of decision trees to enhance reliability metrics in cloud infrastructures. This study aims to bridge this gap by examining the advantages and challenges of using decision trees for reliability in cloud computing. The goal of this study is to provide practical suggestions for cloud service providers to enhance system reliability and effectively reduce operational risks through case studies and real-world scenarios [32].

### Research methodology

The current section provides detailed explanations of the research approach including classification, study design, data collection, exploratory data analysis, data pre-processing, data analysis approaches, & suggested algorithms.

#### Research design

**Fig 1:**
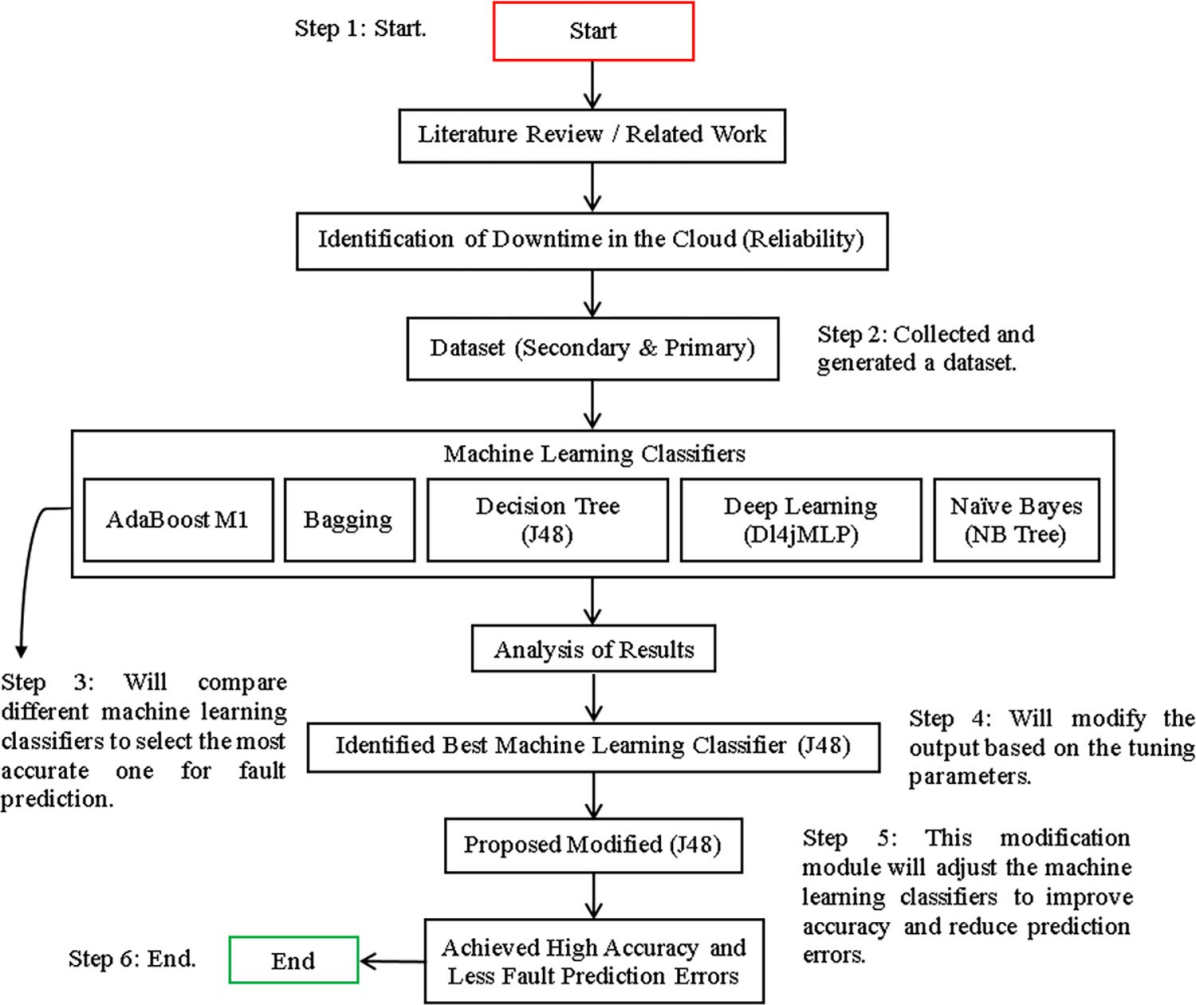
Visualization of the research framework’s implementation view.

The following study design has been followed diligently:

*Proposed model*. In this section, we present our model for fault classification & prediction. The overall research procedure is illustrated in Fig 1. To train our model, we will make use of both main & secondary datasets, while the target datasets will be used for fault classification & prediction. By adopting this approach, we aim to identify the ML classifiers that yield the best outcomes in terms of accuracy, prediction, & data validation by classes, with the minimum possible error in fault prediction.

Furthermore, this section provides a detailed description of the data collection & generation techniques used in the study. The implementation view of the research framework is illustrated in Fig 1.

A solution has been developed to improve accuracy & reduce fault prediction errors in CC using both the primary & secondary datasets Fig 1.

#### Classification

Classification is an ML technique that helps in identifying the condition of a system more accurately & with a lower error rate. It is a supervised learning method to predict a class label for a given sample. The class label (Y) is the category or group that an input variable (X) is transformed into by the output variable (f). For example, determining whether an email is spam or not can be a challenging task for email service providers [15]. Various techniques can be used to obtain the best possible outcome among the four directories of the secondary dataset for classification. We will use a single CSV file to categorize several algorithms for the primary dataset & determine which approach works best for modification.

#### Obtained secondary data

We collected secondary data from the ZENODO website, specifically the Antarex HPC Fault Dataset, which has been used in various studies. This dataset & all details of the testing environment are available to the community for use. Researchers are welcome to utilize the Antarex secondary dataset for ML-based fault prediction studies. The dataset is based on trace data from the Antarex experimental HPC system at ETH Zurich during fault injection [33].

The details of four datasets, namely HDD Mono (3244 instances), HDD Multicores (2493 instances), CPU-Mem Mono (4005 instances), & CPU-Mem Multi (4380 instances) Each instance in these datasets contains eight properties & several occurrences, including timestamp, type, args, seqNum, duration, cores, error, & isFault. These instance types are comprised of numerical & nominal bases. Table 2 provides a summary of the structure of the Antarex dataset.

**Table 2:** Presents an overview of the Antarex dataset structure.

Dataset Directories	Attributes	Attributes Names		Attributes Types	Instances
CPU-Mem Mono	8	1. timestamp	2. type	Numeric & Nominal	4005
CPU- Mem Multi	8	3. args	4. seqNum		4380
HDD Mono	8	5. duration	6. cores		3244
HDD Multi	8	7. error	8. isFault		2493

### Preparing the data for the secondary dataset

Before applying machine learning algorithms to secondary datasets, data pre-processing is required. Three characteristics in this dataset args, seqNum, and duration have duplicate values. Additionally, there are some empty rows and missing values in this collection. Excel’s "Remove Duplicates" function eliminates all duplicate values, as well as empty rows and missing values. After implementing data pre-processing, the secondary dataset displays the information for the following: HDD Mono (Instances: 568), HDD Multicores (Instances: 551), CPU-Mem Mono (Instances: 1740), and CPU-Mem Multi (Instances: 1408).

### Produced primary data

We used the Weibull distribution technique to construct a primary dataset. The Weibull distribution is another commonly used model for predicting the time-to-failure of reliability. It is an extension of the exponential model that allows for non-constant failure rate functions. The Weibull distribution has proven to be effective in explaining both early burnouts & wear-out failures. It is capable of displaying both rising & declining failure rate curves [12]. Different Java platform settings were utilized to generate the primary data using the Weibull distribution method. The parameters used for creating the primary dataset have been summarized in Table 3, whereas Table 4 displays the main dataset.

**Table 3:** A summary of the key parameters responsible for generating the data.

User	Port No	Host No	Network Host	Distribution
1	16	192	Ram, Storage, Bandwidth, and Mips	The Weibull distribution is a probability distribution that models the failure rate of a system over time, and it can represent both increasing and decreasing failure rates.

**Table 4:** A brief synopsis of the main dataset.

FHID	HFTIME	LFT	DIS	DISHT	FTIME/RTIME	STATUS
328	1	-74003	Weibull	0.75:20	11965	Failure
328	1	-74003	Weibull	0.75:20	22765	Repair
453	2	-280036	Weibull	0.75:20	16299	Failure
453	2	-280036	Weibull	0.75:20	27099	Repair
227	1	-133119	Weibull	0.75:20	8498	Failure
227	1	-133119	Weibull	0.75:20	19298	Repair
190	1	-18201	Weibull	0.75:20	7236	Failure
190	1	-18201	Weibull	0.75:20	18036	Repair

The details of the main dataset are displayed in Table 3. The failure host ID (FHID), host failure time (HFT), last failure time (LFT), distribution (Dis), distribution happen time (DHT), failure time/repair time (FTime/RTime), & status are the seven attributes in this core dataset, with a total of 1400 cases. Numerical & nominal bases make up these instance types.

### Machine learning classifiers

This study uses a variety of machine learning-based approaches to anticipate and classify faults. Several classifiers from AdaBoostM1, Bagging, Decision Tree, Deep Learning, and Naive Bayes Tree are used for fault classification and prediction.

### Why is it necessary to compare these machine learning algorithms (AdaBoostM1, Bagging, decision tree (J48), deep learning (DL4JMLP), and naive Bayes)

- These algorithms have different underlying mechanisms, and they perform differently on the same dataset [33].
- AdaBoostM1 combines weak learners in a manner that corrects errors made by previous models, thereby enhancing their accuracy. This iterative process typically results in an overall model with higher accuracy [34].
- Naturally, AdaBoostM1 gives more consideration to harder-to-classify instances, which frequently belong to minority classes in unbalanced datasets. This concentration may enhance the model’s performance on under-represented classes [34].
- By combining predictions from multiple models, bagging reduces the sensitivity of the final prediction to noise in the training set, resulting in more dependable and consistent results [6].
- Neural networks and decision trees are just two examples of the numerous base learners to which bagging can be applied. Its flexibility makes it suitable for a wide variety of machine-learning applications [7].
- J48 is a versatile option for various applications as it can handle both numerical and categorical data, as well as a variety of data formats without requiring further conversion or preparation [7].
- Even with large datasets, J48 can be trained and predicted quickly in most cases because of its efficiency. This makes it suitable for real-time applications or situations requiring a speedy turnaround [6].
- Large datasets can be effectively scaled with DL4JMLP. It can handle massive data volumes and take advantage of parallel processing, both of which are beneficial for big data applications [7].
- Even with large datasets, Naive Bayes is quite efficient in terms of training and prediction times. Due to its simplicity, it can effectively scale to handle larger datasets, making it appropriate for real-time applications [7].

#### AdaBoostM1

One of the most well-known algorithms designed to carry out the general boosting technique is adaptive boosting, which is meant to address binary classification issues. AdaBoostM1 is an expansion of AdaBoost designed to enable it to be used for issues involving more than two classes. Eq 1 illustrates one of its primary features: every poor learner must have an error rate that is equal to or less than 1/2 [34].


$${Pr}_{i∼w1}[h_{f}(x_{i}\neq y_{i})]\leq \sum_{t=1}^{T}\sqrt{1-4Y_{t}^{2}}$$ (1)

#### Bagging

Using several iterations of a predictor to create an aggregated predictor is known as "bagging predictors." When predicting a numerical result, the aggregate averages over the versions; when predicting a class, it uses a plurality vote. By creating multiple bootstrap samples of the training set and using them as new training sets, numerous versions are generated. Bagging has been shown to significantly improve accuracy, as evidenced by tests conducted on real and simulated datasets using regression trees for classification and subset selection in linear regression. The instability of the prediction method is a key factor to consider. In Eq 2 a classification predictor *φ*(*x*, *L*) and predict a class label *j*∈{1,………,*J*} [35].


Q(j|x)=P(∅(x,L)=j (2)

#### Decision tree (J48)

One of the commonly used techniques in various fields such as machine learning, image processing, and pattern recognition is the decision tree. A decision tree is a sequential model that combines a series of basic tests. In each test, a numerical feature is compared to a threshold value. The conceptual principles of decision trees are simpler to establish compared to the numerical weights in the neural network connections between nodes. The primary use of decision trees is for classification. In data mining, the decision tree is a commonly used classification model. Each tree consists of nodes and branches. Each branch specifies a value that can be derived from the node, and each node represents features within a category that require classification [35]. Eqs 3 and 4 show the entropy and information gain [36].


$$Entropy(S)=\sum_{i=1}^{c}P_{i}\;log\;2^{P_{i}}$$ (3)

$$Gain(S,A)={\sum_{v\in }\frac{S_{v}}{S}}_{V(A)}Entropy(S_{v})$$ (4)

#### Deep learning (Dl4jMLP)

Deep learning techniques, which rely on deep neural networks, have recently made significant advancements as a representation of data-driven approaches. Deep neural networks have demonstrated impressive effectiveness in solving a wide range of scientific and technical problems, such as image classification, natural language processing, and defect detection. They are also adept at extracting implicit information of various kinds. According to the universal approximation theorem, a multilayer feedforward network with a sufficient number of hidden layer neurons can approximate any continuous function with arbitrary precision [37].


$$J(0)=\frac{1}{m}\sum_{i=1}^{m}L(y_{i}^{\theta },y_{i})$$ (5)

### Naïve bayes tree

The Naive Bayes algorithm is a supervised machine learning technique that utilizes the conditional probability-based Bayes theorem. It is commonly used for sentiment analysis. This algorithm predicts the text tag and calculates the probability for each tag in a given text, outputting the highest probability [38].

Step 1: Combine a portion of the documents in each class with the probability distribution of P. Word n for class m at word frequency w [38].


$$p(m)\infty \pi_{m}\prod_{n=1}^{\left|v\right|}p(n|m)w_{n}$$ (6)

### Configuring the machine learning classifier parameters

Table 5 displays the various ML classifier parameters with their corresponding values that have been applied to configure ML classifiers to achieve fault prediction by accuracy & class.

**Table 5:** Configuring the ML classifier parameters.

Machine Learning Classifiers	Setting up Parameters	Values
AdaBoostM1	Batch size	100
	Classifier	Decision stump
	Debug	False
	Do not check capabilities	False
	Num decimal places	2
	Num iterations	10
	Resume	False
	Seed	1
	Use resampling	False
	Weight threshold	100
Bagging	Bag size percentage	100
	Batch size	100
	Calc out of bag	False
	Classifier	Rap tree
	Debug	False
	Do not check capabilities	False
	Num decimal places	2
	Num execution slots	1
	Num iterations	10
	Output out of bag complexity statistics	False
	Print classifiers	False
	Represent copies using weights	False
	Seed	1
	Store out of bag predictions	False
Decision Tree (J48)	Batch size	100
	Binary splits	False
	Collapse tree	True
	Confidence factor	0.25
	Debug	False
	Do not check capabilities	False
	Do not make split point actual value	False
	Min num obj	2
	Num decimal places	2
	Num folds	3
	Reduced error pruning	False
	Save instance data	False
	Seed	1
	Sub tree raising	True
	Unpruned	False
	Use la place	False
	Use MDL correction	True
Deep Learning (Dl4jMLP)	Log config	Log configuration
	Layer specification	1 weka.dl4j.layers.layer
	Preview zoo model layer spec in GUI	False
	Number of epochs	10
	Instance iterator	Default instance iterator
	Early stopping	Early stopping
	Network configuration	Neural net configuration
	Set the iteration listener	Epoch listener
	Zoo model	Custom net
	Attribute normalization	Standardize training data
	Set the cache mode	Memory
	Data queue size	0
	Resume	False
	Preserve file system cache	False
	Number of GPUs	1
	Size of per fetch buffer for multiple GPUs	24
	Model parameter averaging frequency	10
	Batch size	100
	Debug	False
	Do not check capabilities	False
	Num decimal places	2
	Seed	1
Naïve Bayes Tree (NBTree)	Batch size	100
	Debug	False
	Do not check capabilities	False
	Num decimal places	2

### Modified decision tree (J48)

**Fig 2:**
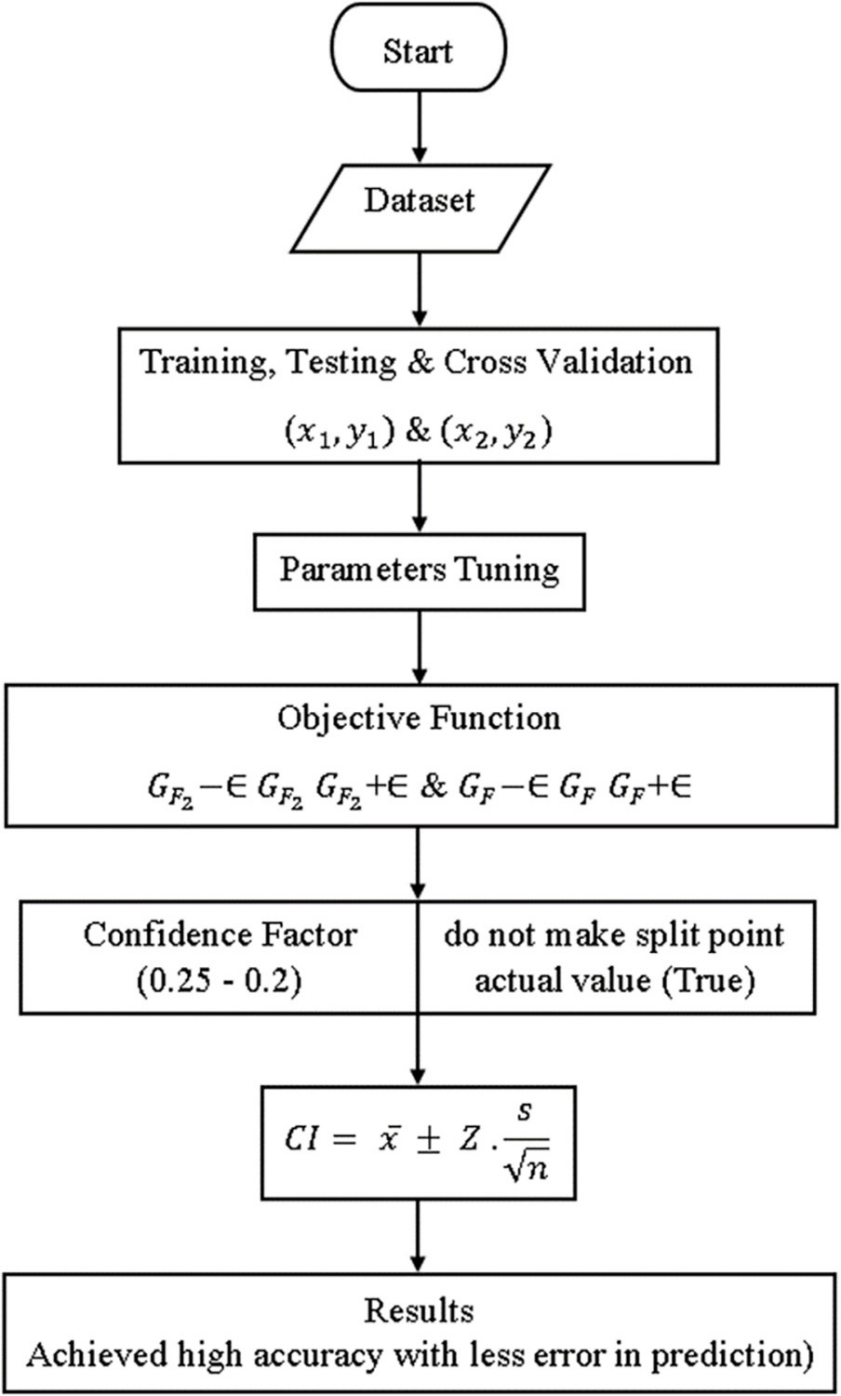
Shows the block diagram of the modified decision tree (J48) classifier.

The original J48 method suffers from poor accuracy & a high rate of fault prediction errors. To address these issues, this research aims to use a modified decision tree (J48), which achieves higher accuracy while making fewer prediction errors. The block diagram of the modified decision tree (J48) classifier is shown in Fig 2. High accuracy & less fault prediction errors are predicated on the created primary dataset. Using an objective function, high accuracy & low fault prediction error have been assessed for $G_{F_{2}}-\in G_{F_{2}}\;G_{F_{2}}+\in$ and $G_{F}-\in G_{F}\;G_{F}+\in$. High precision & reduced error in fault prediction has been achieved by utilizing objective functions via algorithm parameters. The confidence factor parameter ranges from 0.25 to 0.1, and do not make the split point actual value (true).

The mathematical formula for modified decision tree (J48) classifiers is displayed in Eqs 7–9 below.


$$\left(x_{1},y_{1})\;and\;(x_{2},y_{2}\right)$$ (7)

Eq 7 defines the terms training, testing, and cross-validation.


$$G_{F_{2}}-\in G_{F_{2}}\;G_{F_{2}}+\in \&G_{F}-\in G_{F}\;G_{F}+\in$$ (8)

The objective function is defined by Eq 8.

$$CI=\bar{x}\pm Z.\frac{s}{\sqrt{n}}$$ (9)

The confidence factor and do not make split point actual value in Eq 9 are defined as above.

## Experiments and results

This section showcases the data analysis & classification results obtained from various ML techniques including AdaBoostM1, Bagging, J48, Dl4jMLP, & NBTree with a confusion matrix. The primary focus of this investigation is the Modified Decision Tree (J48), and its findings are presented below. This research aims to utilize traditional ML techniques to minimize fault prediction errors & achieve high levels of accuracy.

The secondary dataset archive contains four folders, each for a different dataset block: CPU/Memory & HDD in both single-core & multi-core versions [11]. The analysis of the results indicates a significant difference between the four directories of the secondary dataset. The CPU-Mem Multi cores folder outperforms the other directories, which include CPU-Mem Mono, HDD Mono, & HDD Multi.

The primary dataset performs better than the secondary dataset, based on the comparisons, therefore in this study, the primary dataset results were enough to take into account when adjusting the ML algorithm.

The data was trained using AdaBoostM1, Bagging, J48, Dl4jMLP, & NBTree classifiers with 80/20, 70/30, & 10-fold cross-validation, & successfully obtained the necessary classification results for both Secondary & Primary categories. Eqs 10 through 20 were used to measure data validation, fault prediction error, & accuracy by class to evaluate the performance of these classifiers. The results from a secondary dataset (CPU-Mem Multi) indicated that J48 outperformed AdaBoostM1, Bagging, Dl4jMLP, & NBTree. On the other hand, the primary dataset’s results showed that NBTree performed better, although it had poor time complexity. Based on the primary dataset, we found that there are some minor differences in point values between NBTree & J48. However, J48 has a good temporal complexity. We performed our analysis using WEKA 3.8.6 software environment, with the Remove Percentage Filter enabled.


$$Accuracy=\;\frac{\mathrm{TP}+\mathrm{TN}}{\mathrm{TP}+\mathrm{TN}+\mathrm{FP}+\mathrm{FN}}$$ (10)

In Eq 10 the accuracy is defined as above.


$$Recall\;or\;True-Positive\;Rate=\;\frac{\mathrm{TP}}{\mathrm{TP}+\mathrm{FN}}$$ (11)

In Eq 11 the recall or true positive rate is defined as above.


$$True‐Negative\;Rate=\;\frac{\mathrm{TN}}{\mathrm{TN}+\mathrm{FP}}$$ (12)

In Eq 12 the true negative rate is defined as above.


$$Precision=\;\frac{\mathrm{TP}}{\mathrm{TP}+\mathrm{FP}}$$ (13)

In Eq 13 the precision is defined as above.


$$False‐Positive\;Rate=\;\frac{\mathrm{FP}}{\mathrm{TN}+\mathrm{FP}}$$ (14)

In Eq 14 the false positive rate is defined as above.


$$MCC=\;\frac{\mathrm{TP}.\mathrm{TN}-\mathrm{FP}.\mathrm{FN}}{\surd (\mathrm{TP}+\mathrm{FP})(\mathrm{TP}+\mathrm{FN})(\mathrm{TN}+\mathrm{FP})(\mathrm{TN}+\mathrm{FN})}$$ (15)

In Eq 15 the Matthews correlation coefficient is defined as above.


$$F‐Measure=\;\frac{2\mathrm{PPV}\times \mathrm{TPR}}{\mathrm{PPV}+\mathrm{TPR}}$$ (16)

In Eq 16 the F-measure is defined as above.

- A commonly used method to compare predicted & observed values of a model or estimator is the root mean square error (RMSE) [39].
- Two different continuous variables are being measured using the Mean Absolute Error (MAE) method [39].
- Normalizing the total absolute error, relative absolute error is obtained by dividing the total absolute error by the total absolute error of the simple predictor [40].
- Normalizing the total squared error, the relative squared error (RSE) is obtained by dividing the former by the total squared error of the simple predictor [40].

$$RMSE=\sqrt{\frac{1}{n}\sum_{i=1}^{n}\left(y_{i}-\hat{yi}\right)}$$ (17)

In Eq 17 the RMSE is defined as above.


$$MAE=\frac{1}{n}\sum_{i=1}^{n}\left|y_{i}-\hat{y_{i}}\right|$$ (18)

In Eq 18 the MAE is defined as above.


$$E_{i}=\frac{\sum_{j=1}^{n}\left|P_{\left(ij\right)}-T_{j}\right|}{\sum_{j=1}^{n}\left|T_{j}-\bar{T}\right|}$$ (19)

In Eq 19 the RAE is defined as above.


$$E_{i}=\sqrt{\frac{\sum_{j=1}^{n}(P_{\left(ij\right)}-T_{j}{)}^{2}}{\sum_{j=1}^{n}(T_{j}-\bar{T}{)}^{2}}}$$ (20)

In Eq 20 the RSE is defined as above.

### 1. Comparing various classification methods using a secondary dataset

We evaluated various classifiers using ISFAULT with secondary data & STATUS with main data. Our models included AdaBoostM1, Bagging, J48, Dl4jMLP, & NBTree.

**Fig 3:**
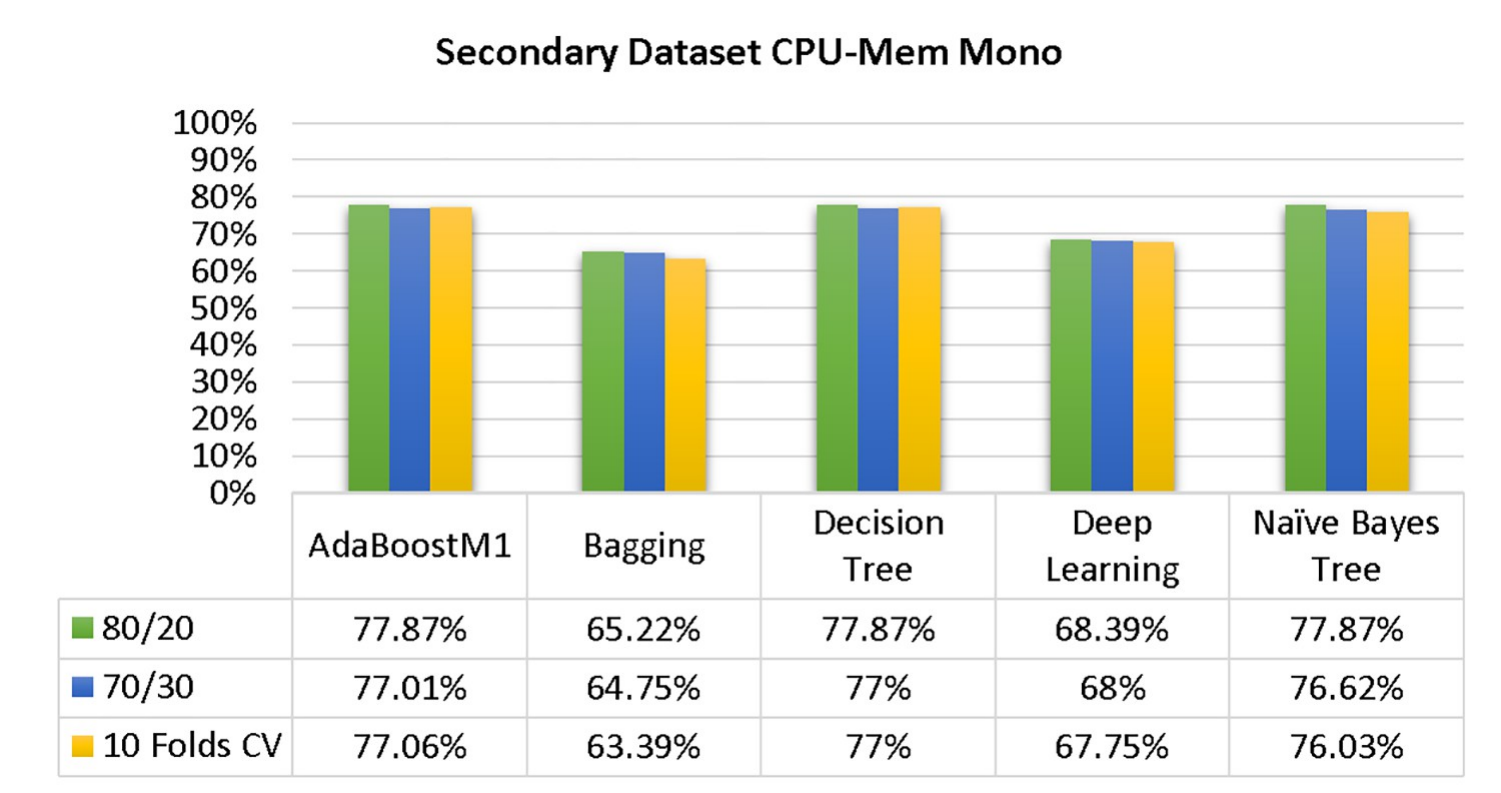
Shows the accuracy of CPU-mem mono on ML classifiers for each class (true/false).

**Fig 4:**
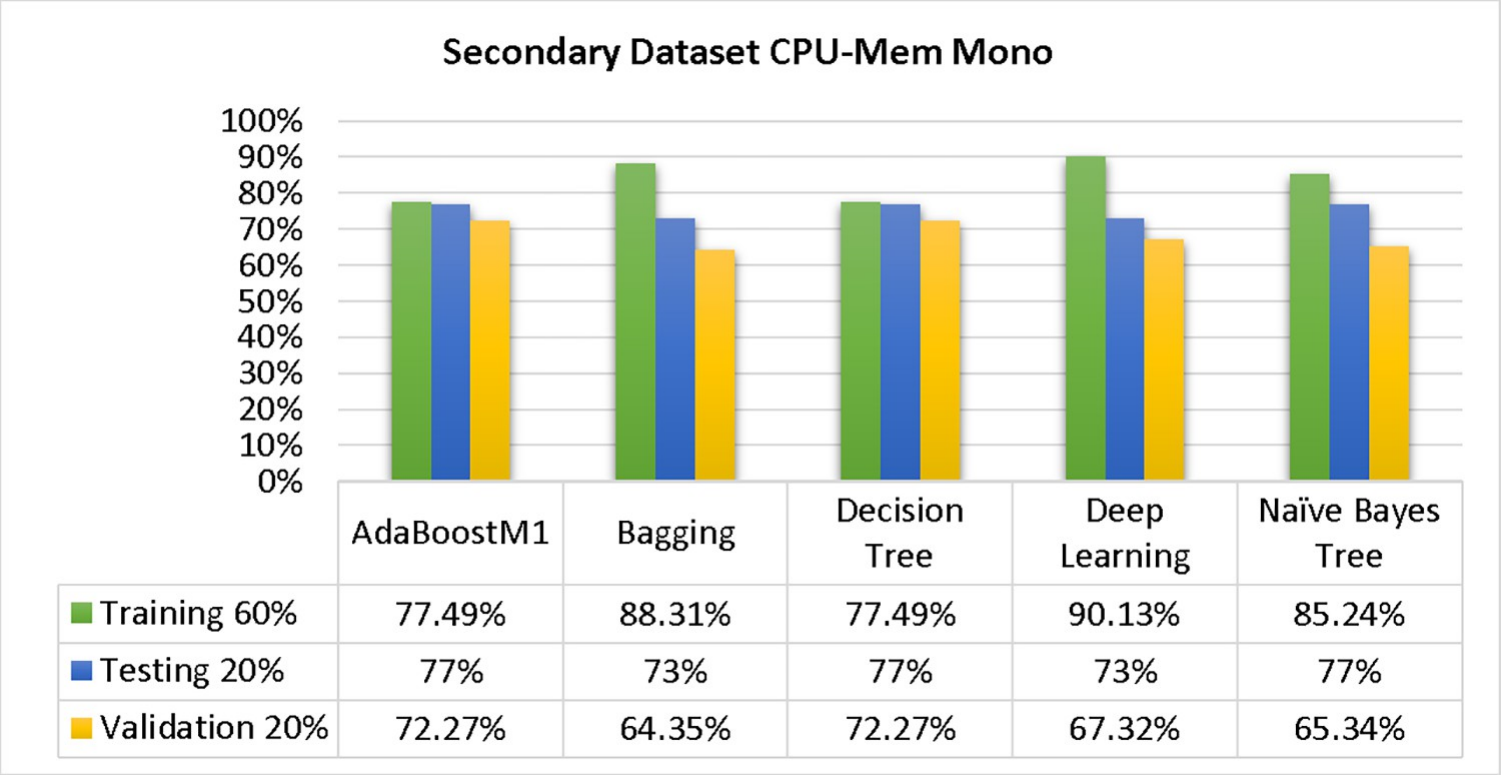
Shows CPU-mem mono class (true/false) ML classifiers’ accuracy regarding data validation outcomes.

**Fig 5:**
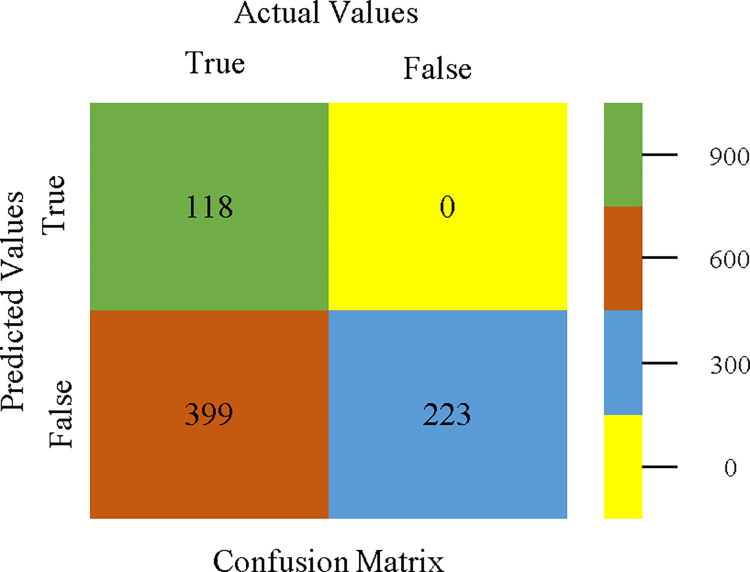
AdaBoostM1 classifier’s confusion matrix for accuracy & fault prediction based on CPU-mem mono.

**Fig 6:**
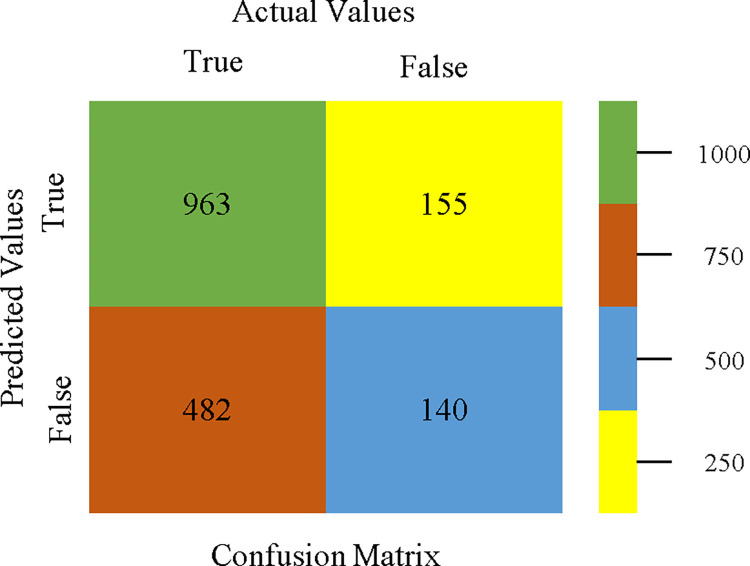
Bagging classifier’s confusion matrix for accuracy & fault prediction based on CPU-mem mono.

**Fig 7:**
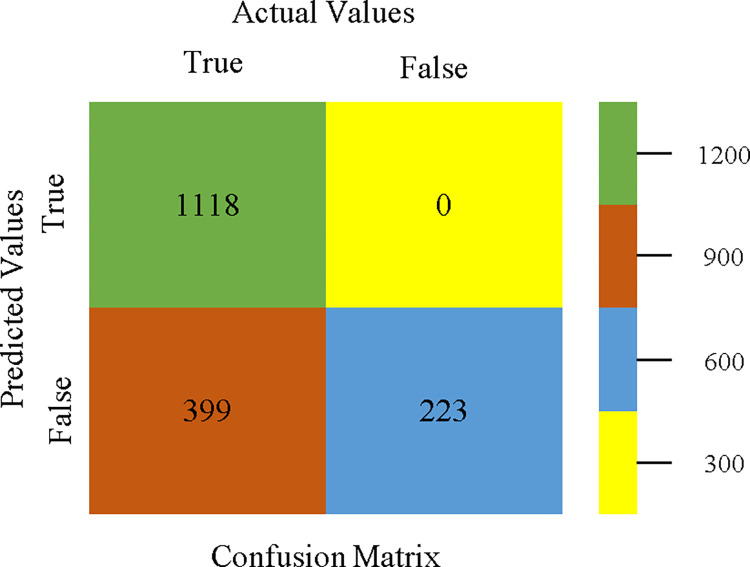
J48 classifier’s confusion matrix for accuracy & fault prediction based on CPU-mem mono.

**Fig 8:**
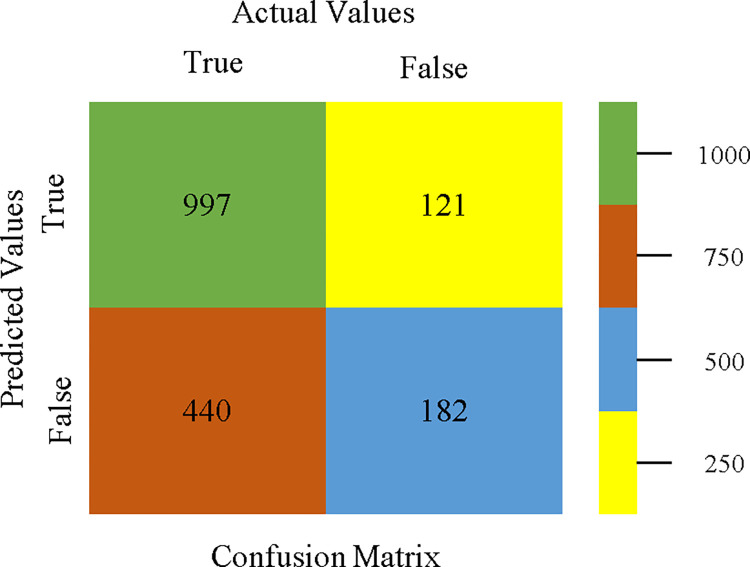
Dl4jMLP classifier’s confusion matrix for accuracy & fault prediction based on CPU-mem mono.

**Fig 9:**
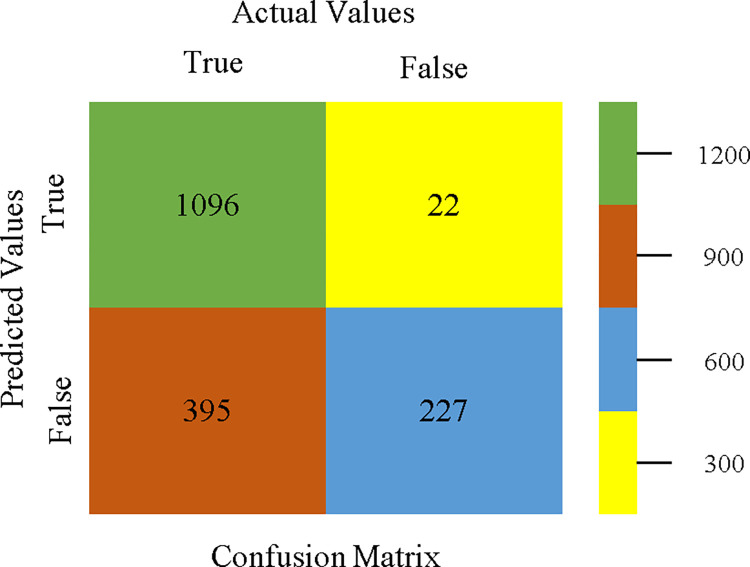
NBTree classifier’s confusion matrix for accuracy & fault prediction based on CPU-mem mono.

**Fig 10:**
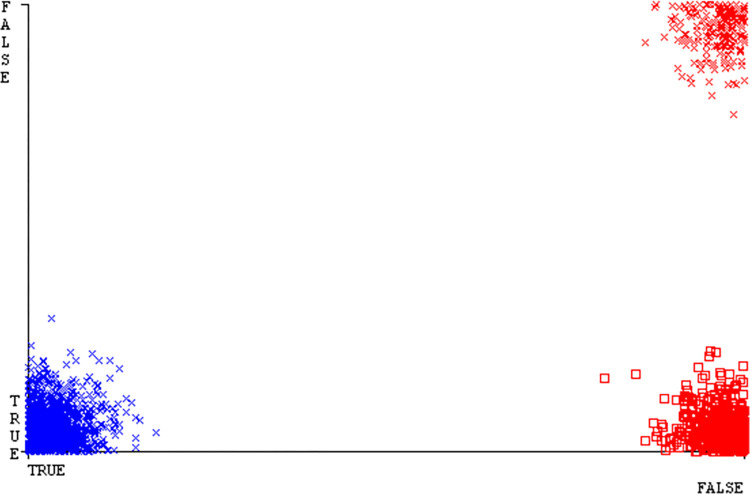
Classifier errors of AdaBoostM1 based on CPU-mem mono in accuracy & fault prediction.

**Fig 11:**
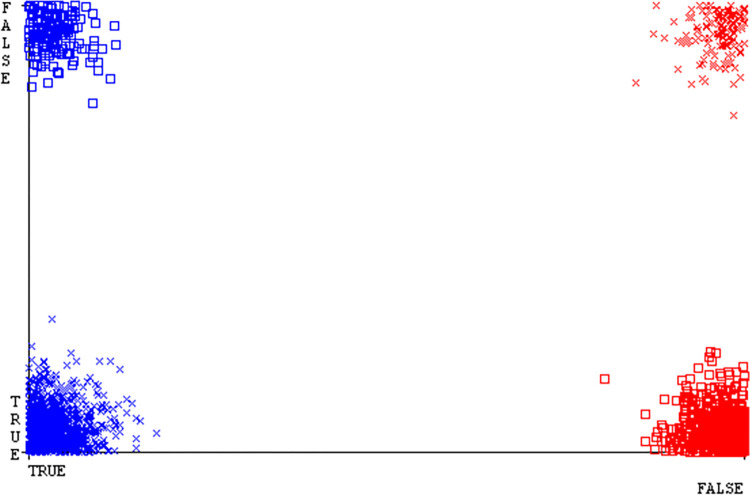
Classifier errors of Bagging based on CPU-mem mono in accuracy & fault prediction.

**Fig 12:**
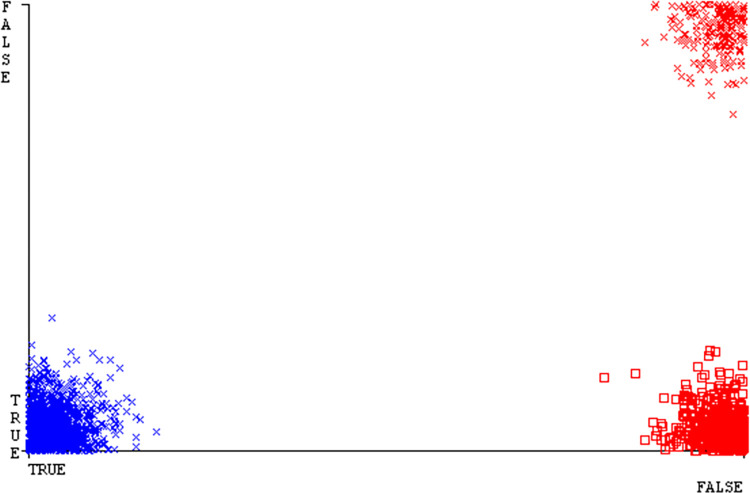
Classifier errors of J48 based on CPU-mem mono in accuracy & fault prediction.

**Fig 13:**
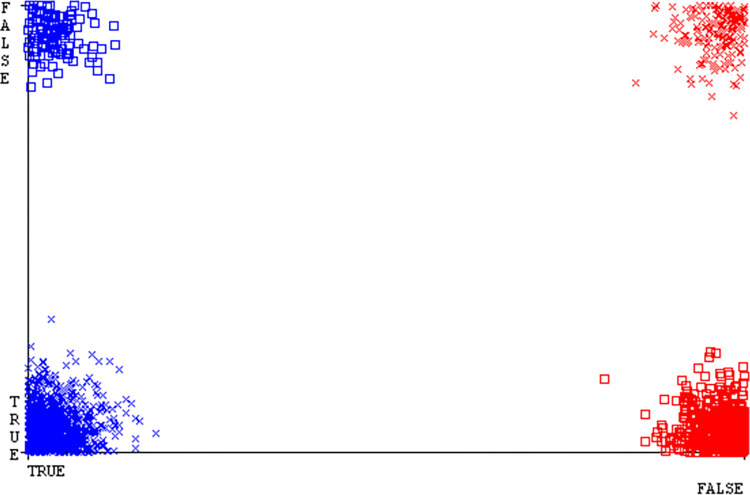
Classifier errors of Dl4jMLP based on CPU-mem mono in accuracy & fault prediction.

**Fig 14:**
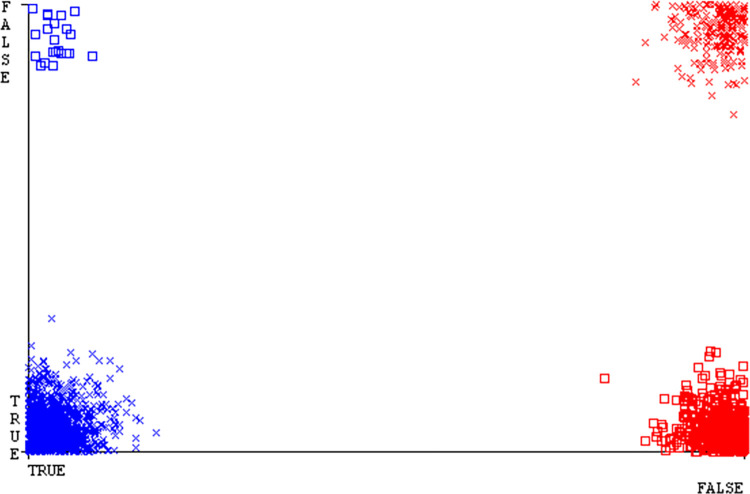
Classifier errors of NBTree based on CPU-mem mono in accuracy & fault prediction.

**Fig 15:**
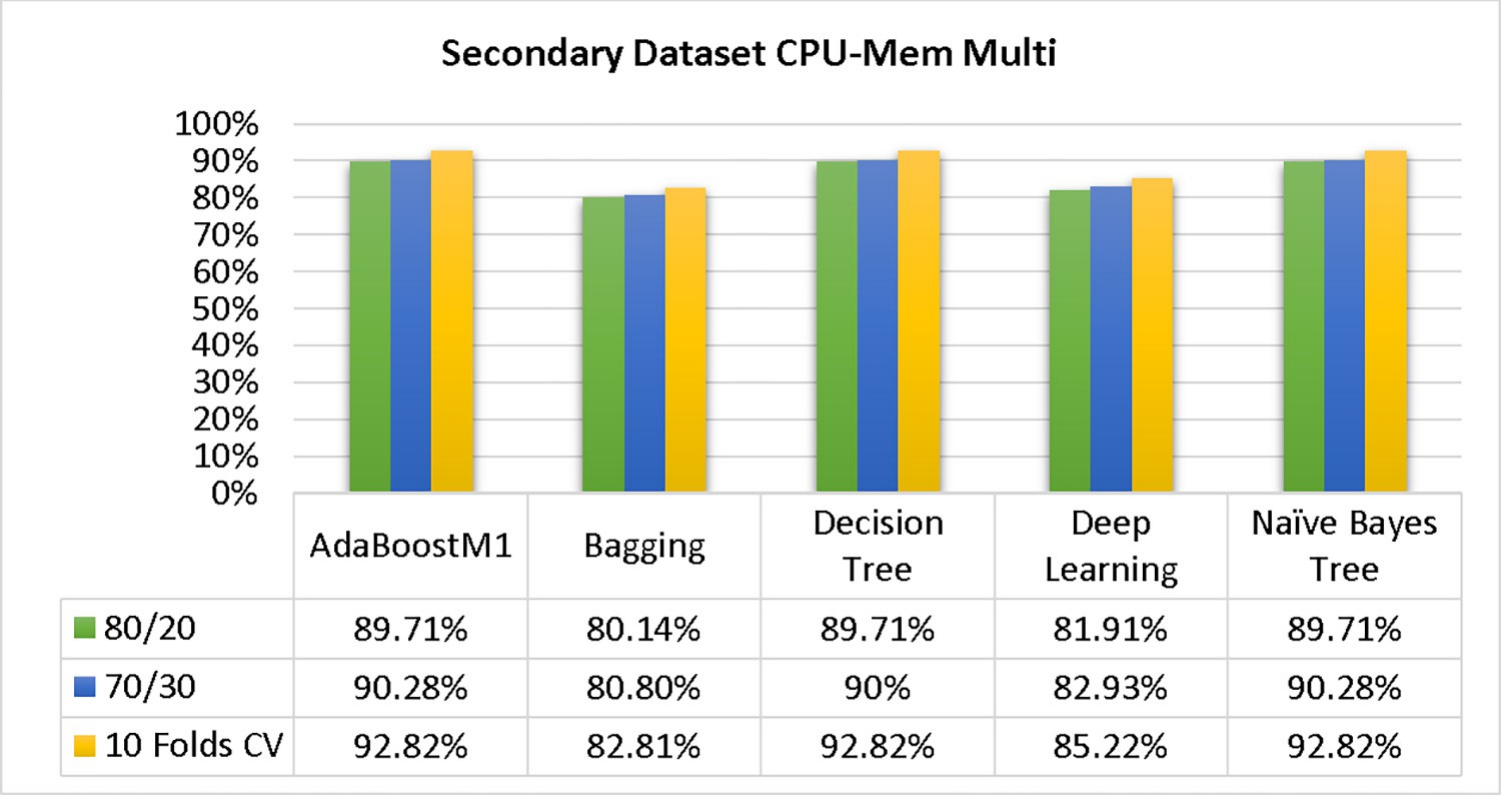
Shows the accuracy of CPU-mem multi on ML classifiers for each class (true/false).

**Fig 16:**
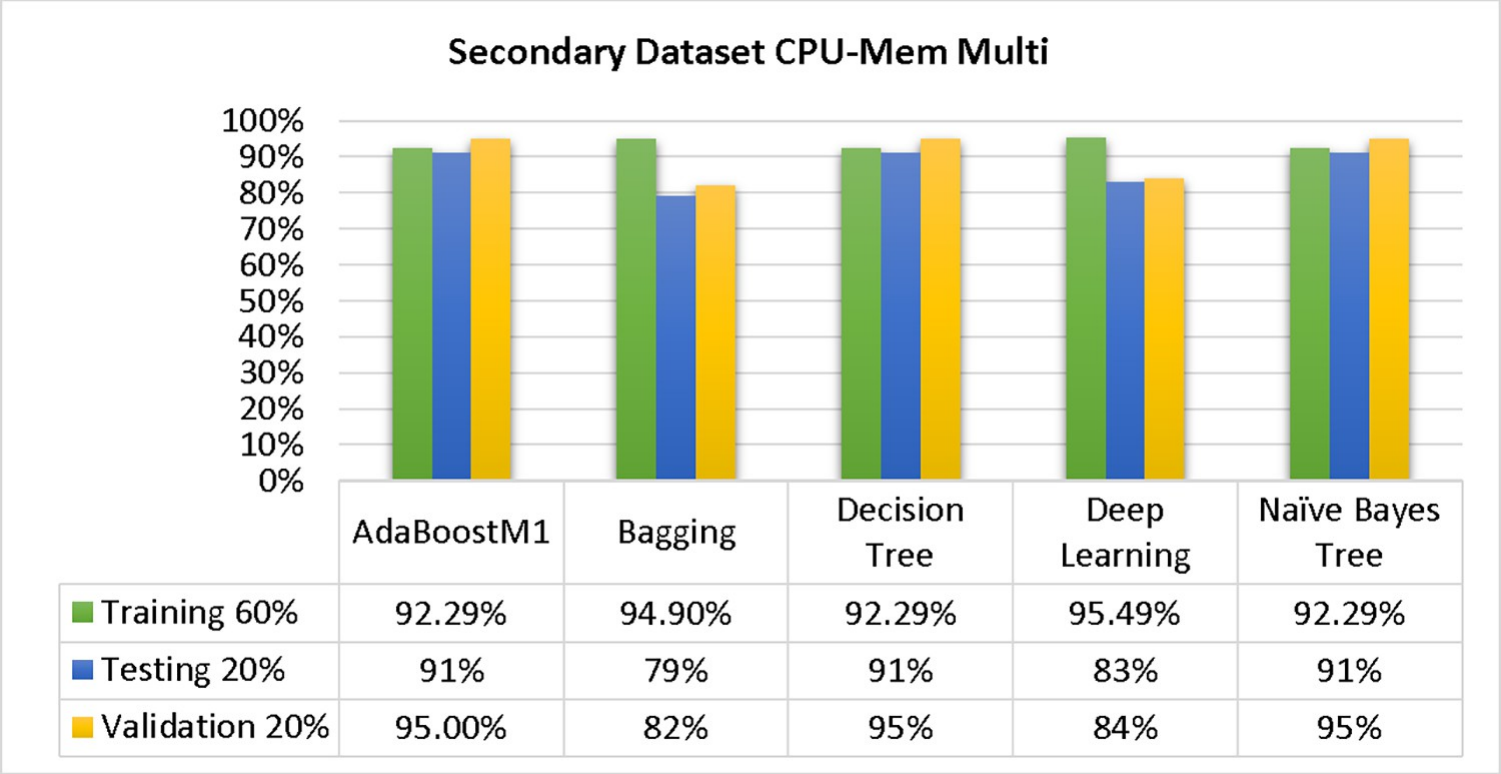
Shows CPU-mem multi-class (true/false) ML classifiers’ accuracy regarding data validation outcomes.

**Fig 17:**
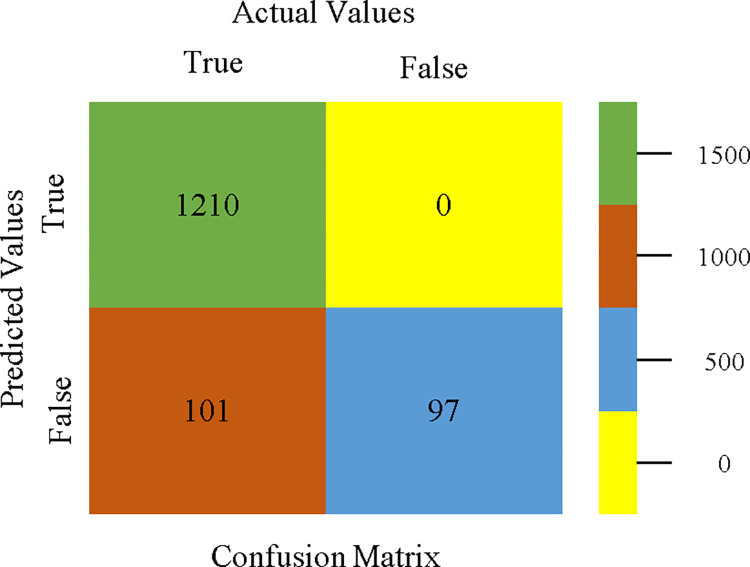
AdaBoostM1 classifier’s confusion matrix for accuracy & fault prediction based on CPU-mem multi.

**Fig 18:**
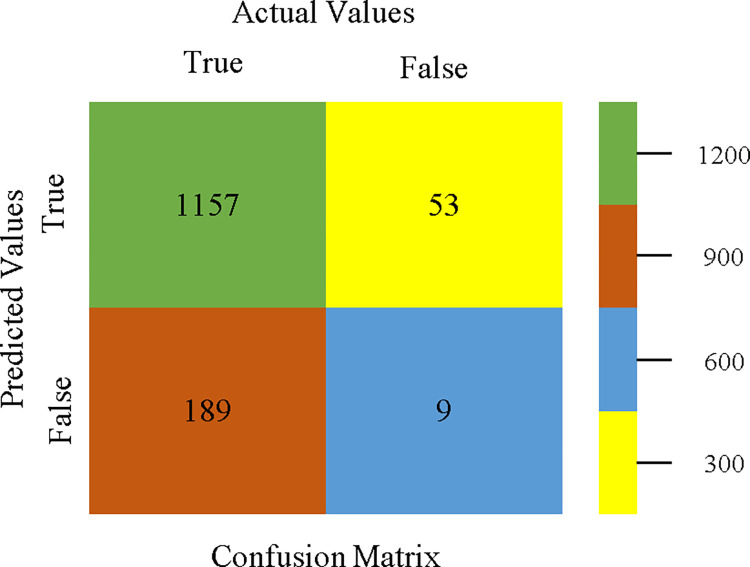
Bagging classifier’s confusion matrix for accuracy & fault prediction based on CPU-mem multi.

**Fig 19:**
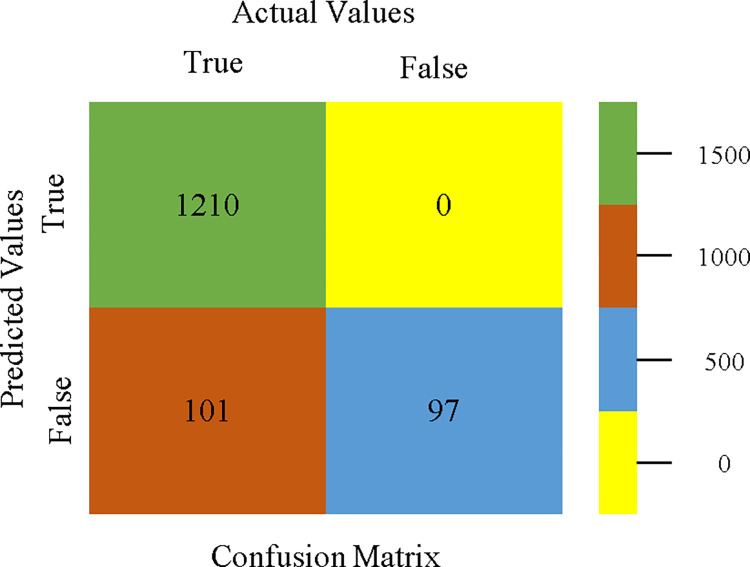
J48 classifier’s confusion matrix for accuracy & fault prediction based on CPU-mem multi.

**Fig 20:**
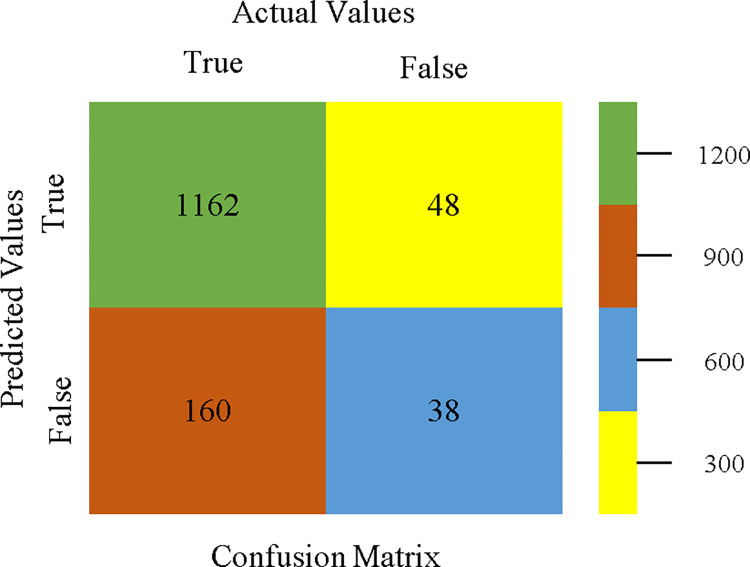
Dl4jMLP classifier’s confusion matrix for accuracy & fault prediction based on CPU-mem multi.

**Fig 21:**
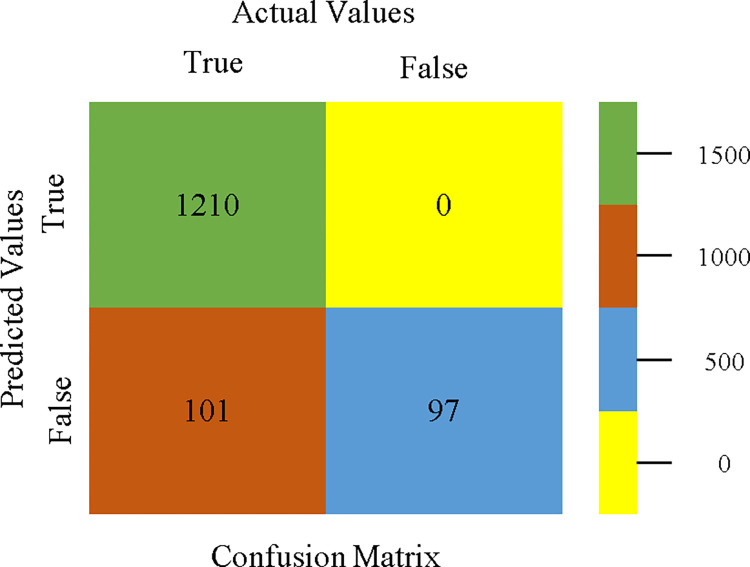
NBTree classifier’s confusion matrix for accuracy & fault prediction based on CPU-mem multi.

**Fig 22:**
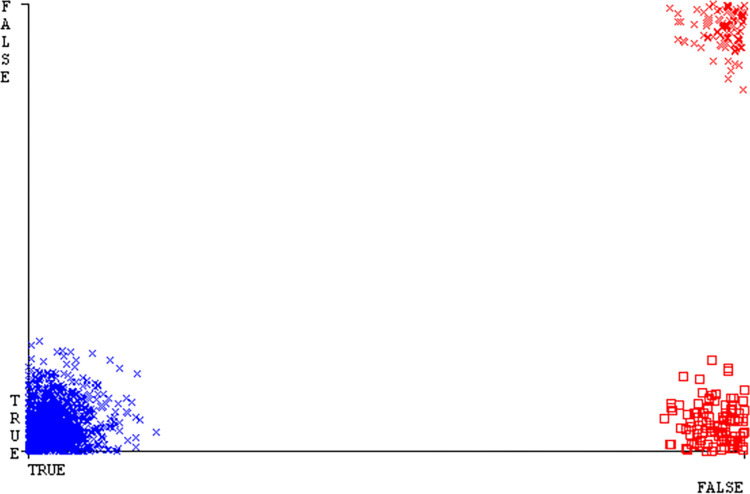
Classifier errors of AdaBoostM1 based on CPU-mem multi in accuracy & fault prediction.

**Fig 23:**
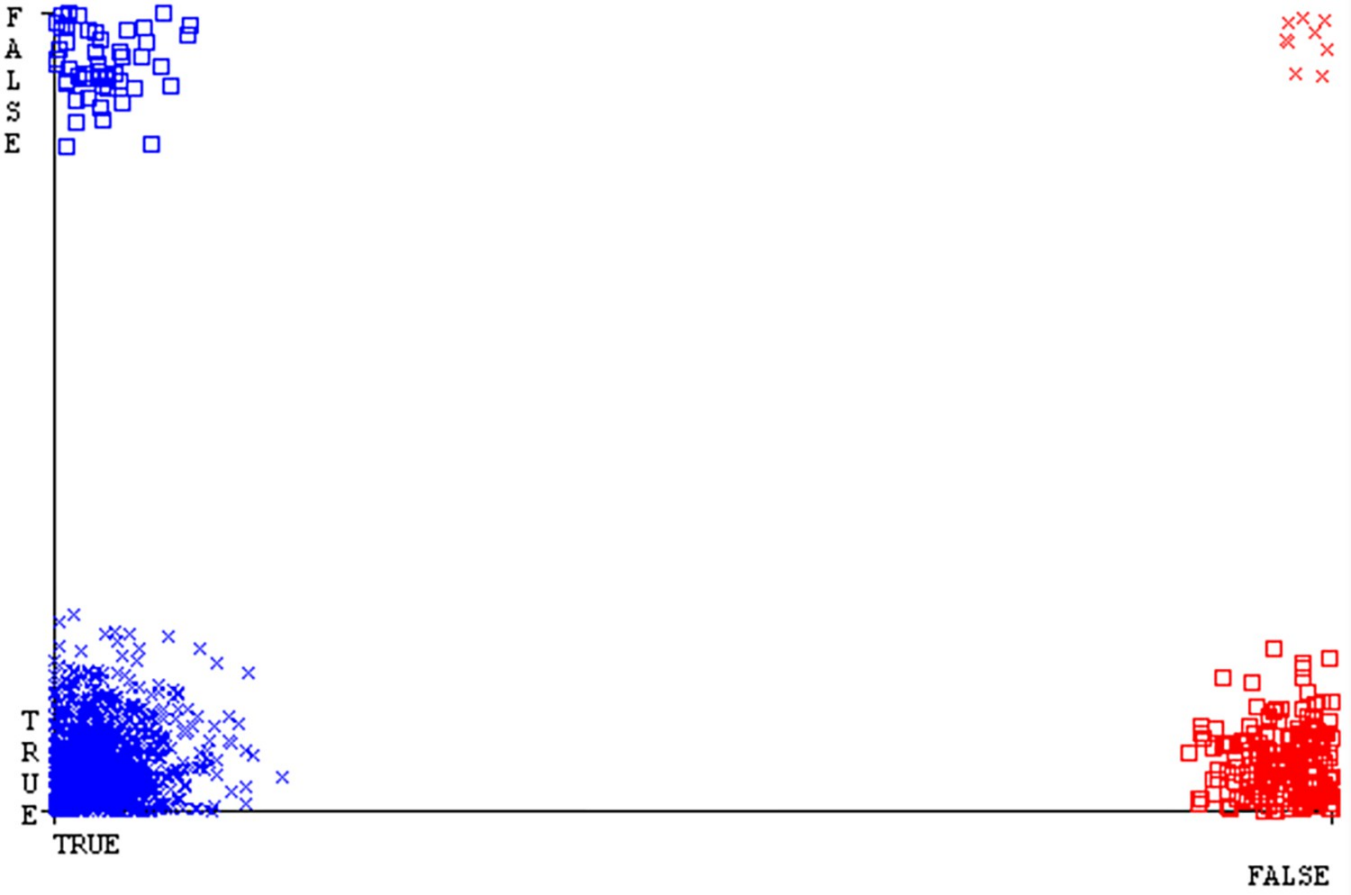
Classifier errors of Bagging based on CPU-mem multi in accuracy & fault prediction.

The results of each classifier’s secondary & primary data using different cross-validation techniques are shown in Figs 3–50. The results demonstrate excellent accuracy & low fault prediction. 60% of the data is used for training, 20% for testing, & 20% for validation. Among the secondary data results, CPU-Mem Multi has the highest accuracy & the least amount of fault prediction on the J48 classifier using 80/20 (89.71%), 70/30 (90.28%), & 10-fold cross-validation (92.82%). Similarly, HDD-Mono yields 80/20 (90.35%), 70/30 (92.35%), & 10-fold cross-validation (90.49%). Based on the results of 80/20 (93.63%), 70/30 (90.09%), & 10 folds cross-validation (88.92%) on HDD Multi, the AdaBoostM1 classifier provides the highest level of accuracy & the least amount of fault prediction. Similarly, on CPU-Mem Mono, the AdaBoostM1 classifier has shown the highest accuracy percentage of 77.87% for 80/20, 77.01% for 70/30, & 77.06% for 10-fold cross-validation.

**Fig 24:**
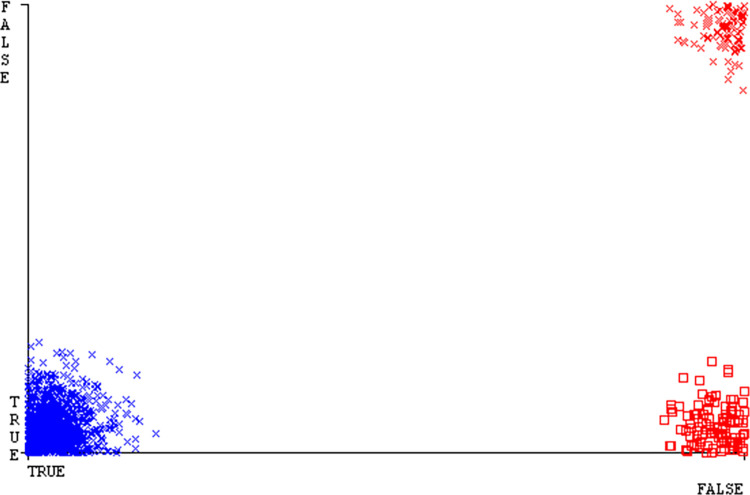
Classifier errors of J48 based on CPU-mem multi in accuracy & fault prediction.

**Fig 25:**
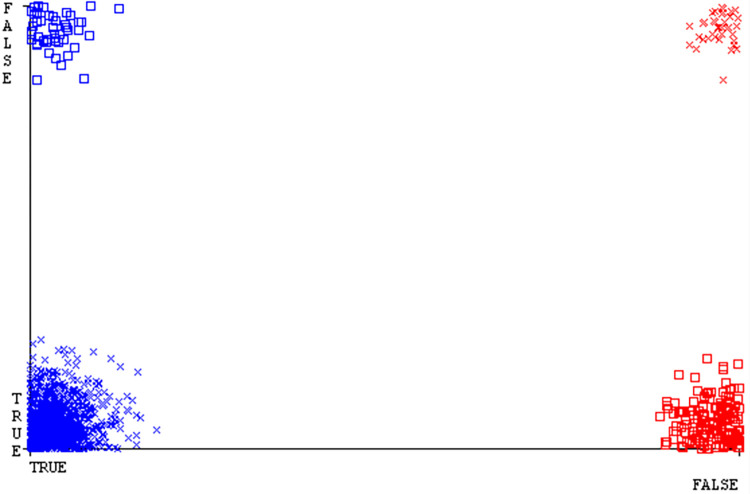
Classifier errors of Dl4jMLP based on CPU-mem multi in accuracy & fault prediction.

**Fig 26:**
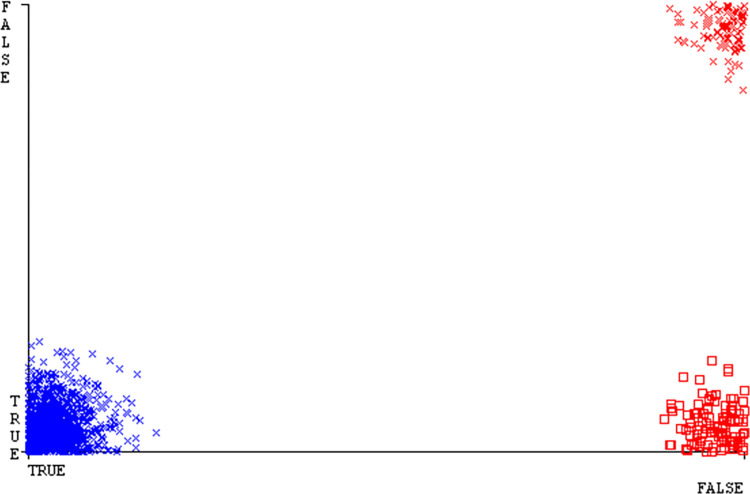
Classifier errors of NBTree based on CPU-mem multi in accuracy & fault prediction.

**Fig 27:**
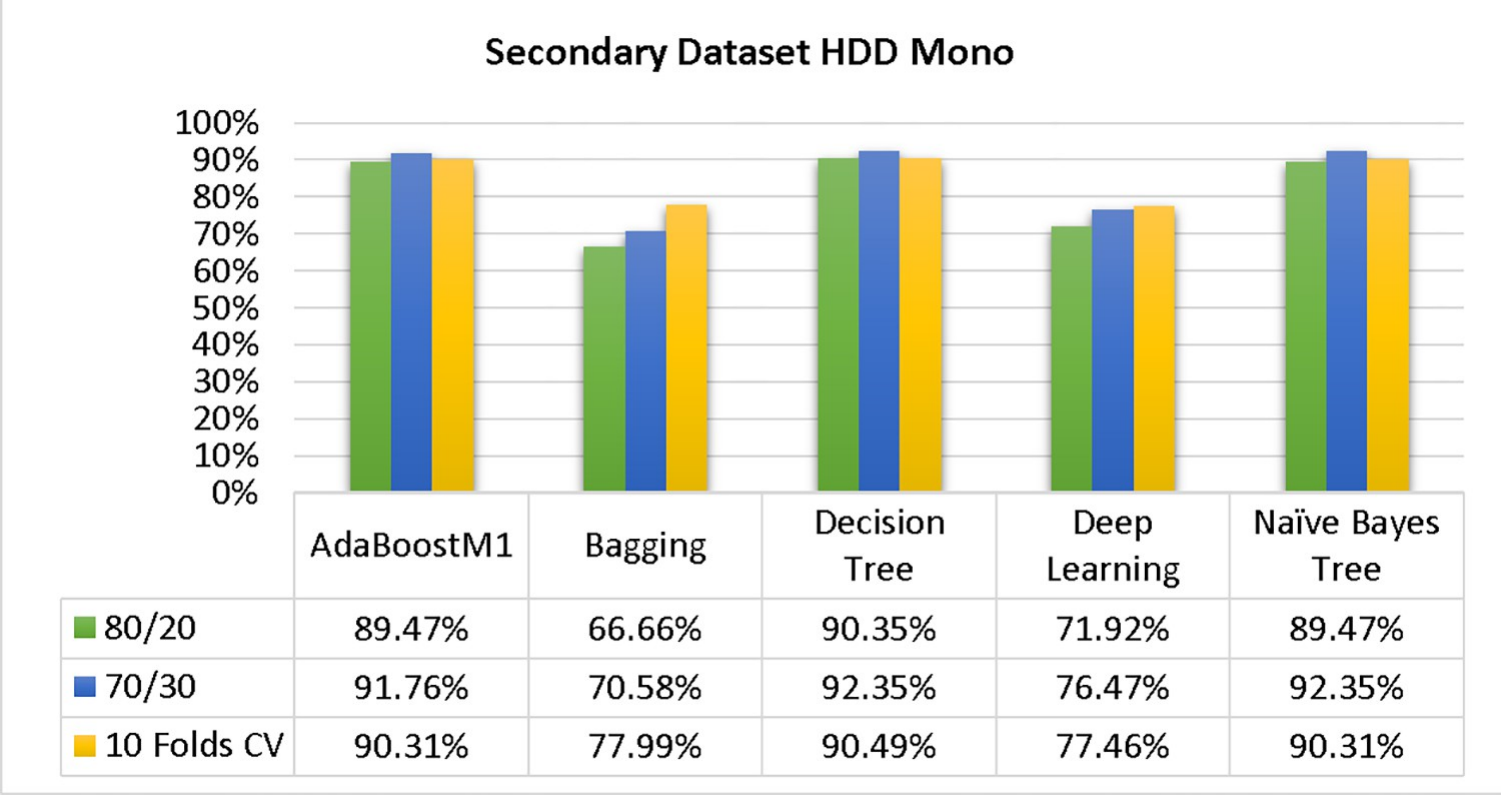
Shows the accuracy of HDD Mono on ML classifiers for each class (true/false).

**Fig 28:**
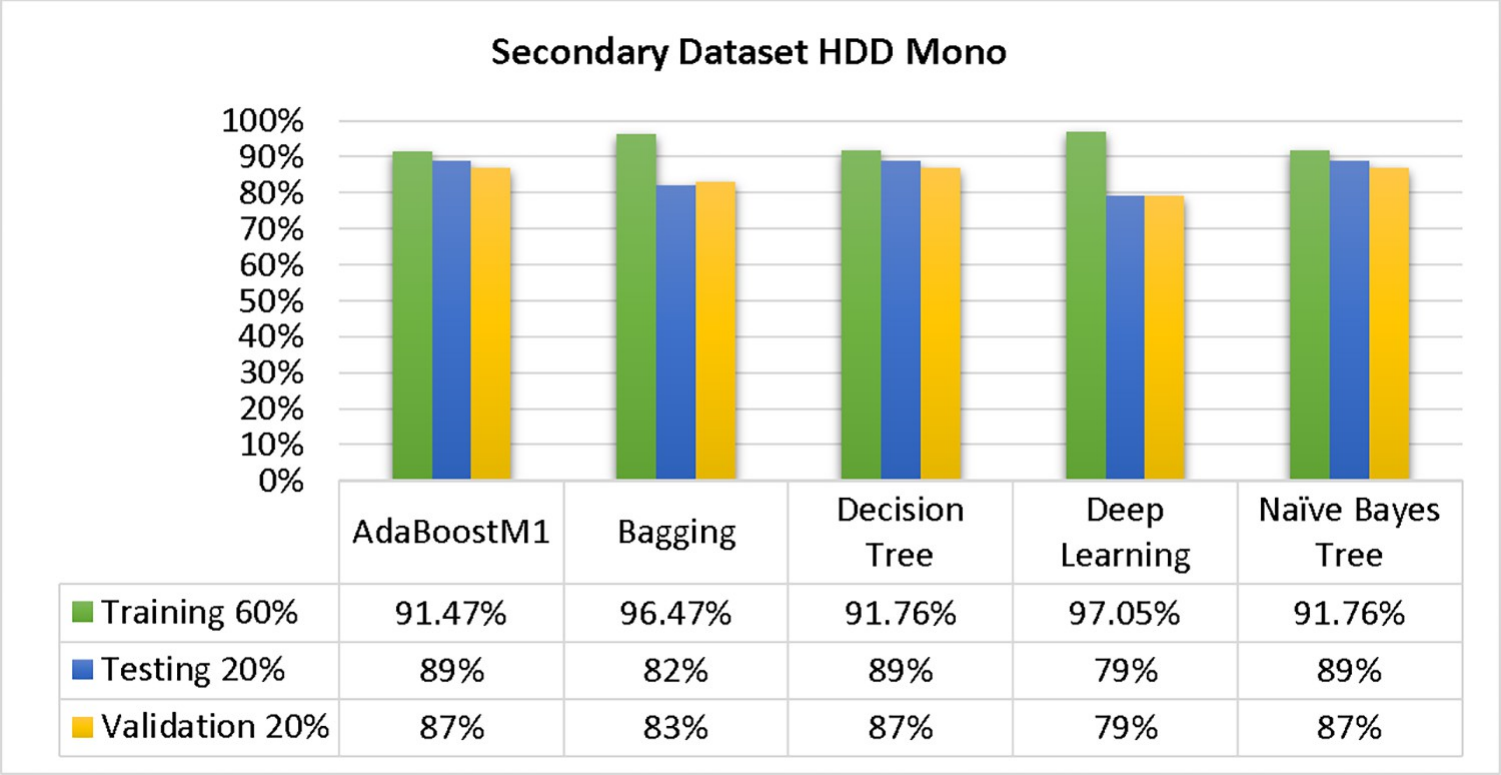
Shows HDD Mono class (true/false) ML classifiers’ accuracy regarding data validation outcomes.

**Fig 29:**
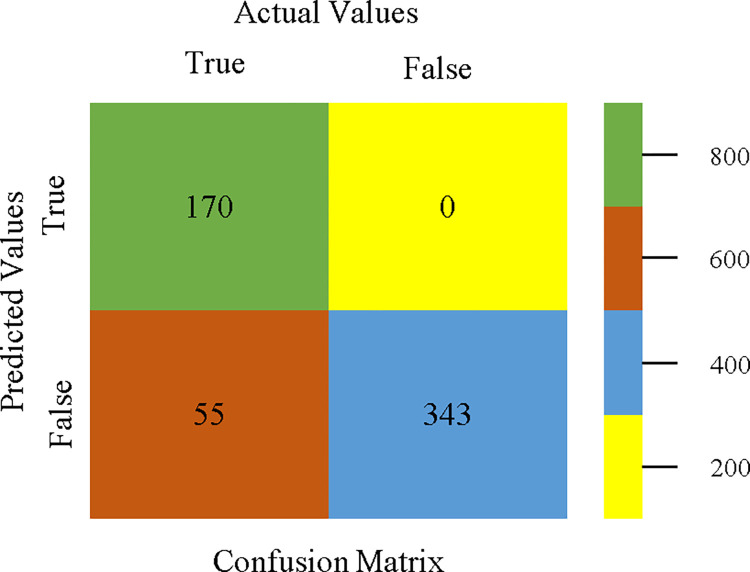
AdaBoostM1 classifier’s confusion matrix for accuracy & fault prediction based on HDD mono.

**Fig 30:**
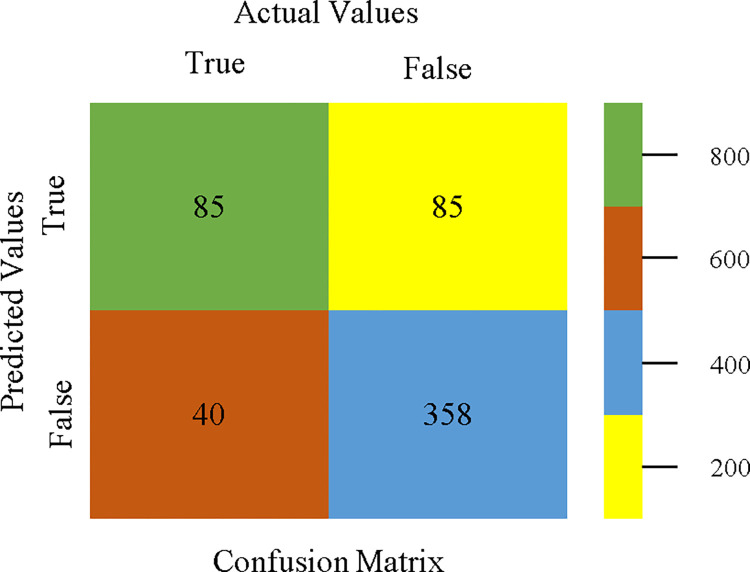
Bagging classifier’s confusion matrix for accuracy & fault prediction based on HDD mono.

**Fig 31:**
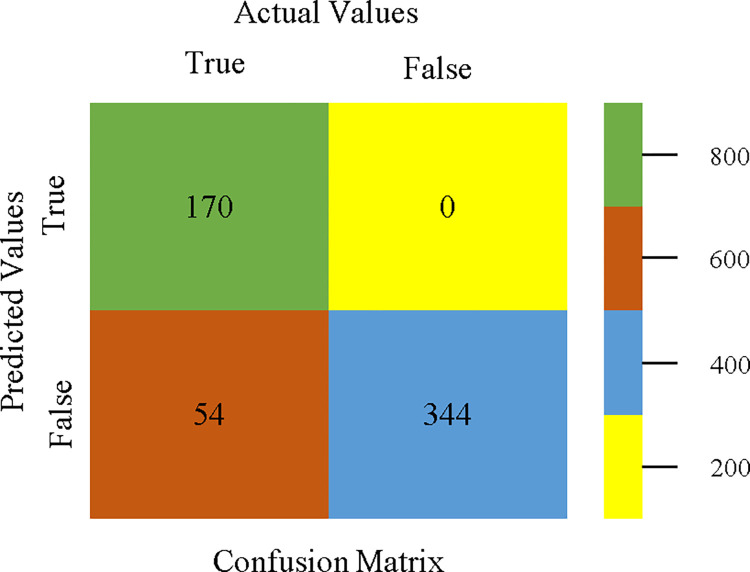
J48 classifier’s confusion matrix for accuracy & fault prediction based on HDD mono.

**Fig 32:**
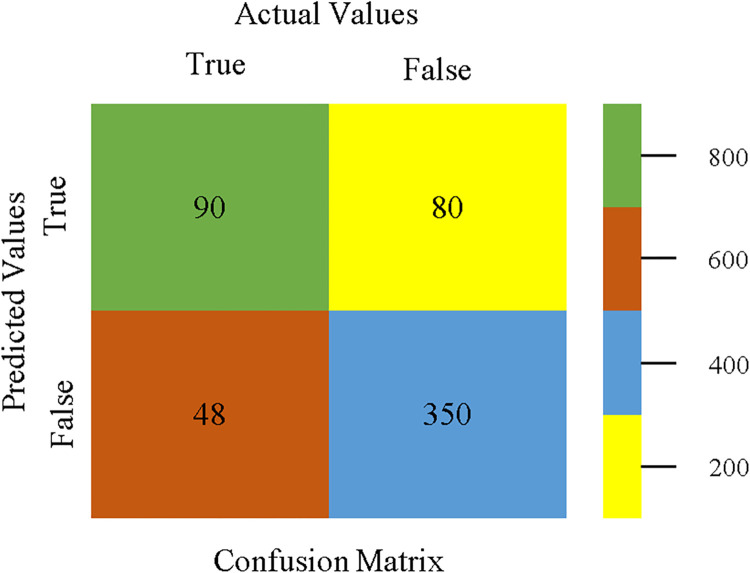
Dl4jMLP classifier’s confusion matrix for accuracy & fault prediction based on HDD mono.

**Fig 33:**
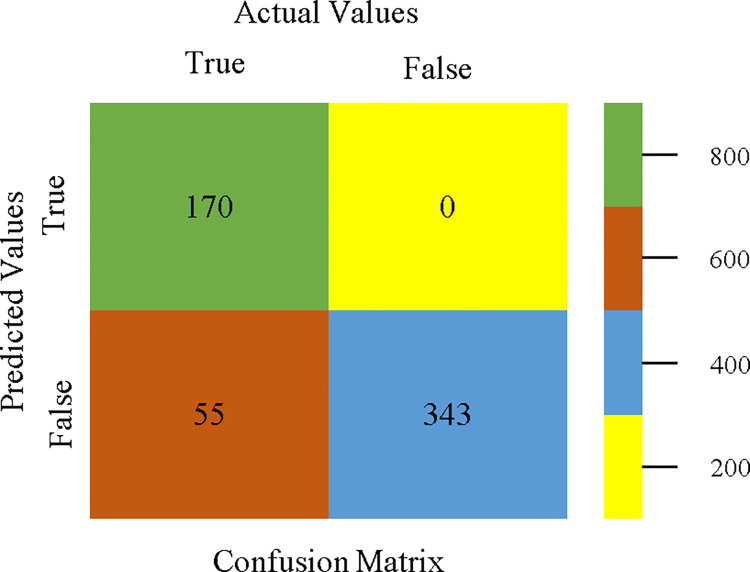
NBTree classifier’s confusion matrix for accuracy & fault prediction based on HDD mono.

**Fig 34:**
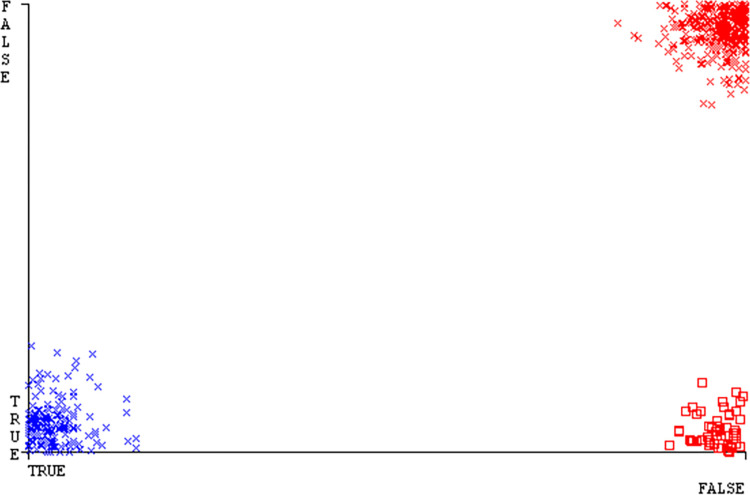
Classifier errors of AdaBoostM1 based on HDD mono in accuracy & fault prediction.

**Fig 35:**
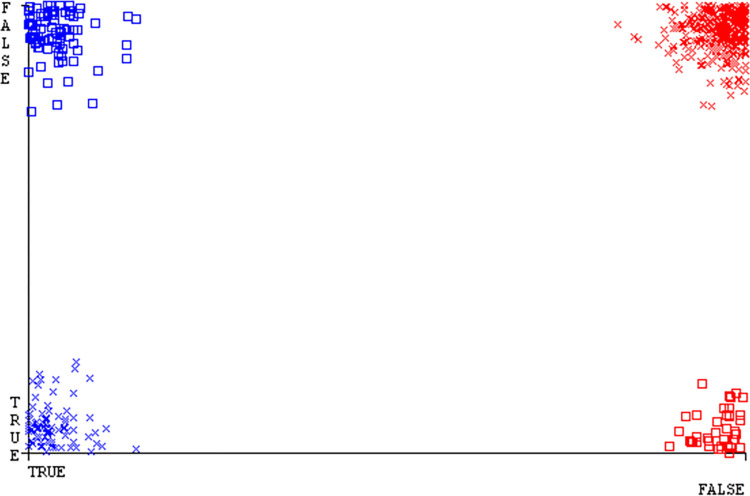
Classifier errors of Bagging based on HDD mono in accuracy & fault prediction.

**Fig 36:**
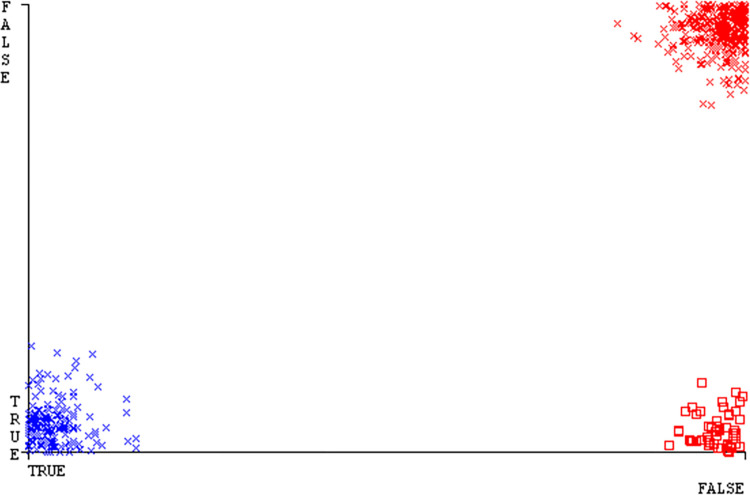
Classifier errors of J48 based on HDD mono in accuracy & fault prediction.

**Fig 37:**
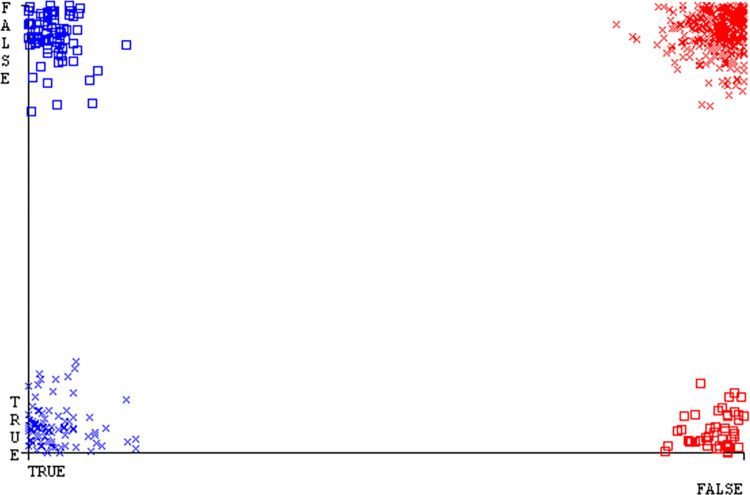
Classifier errors of Dl4jMLP based on HDD mono in accuracy & fault prediction.

**Fig 38:**
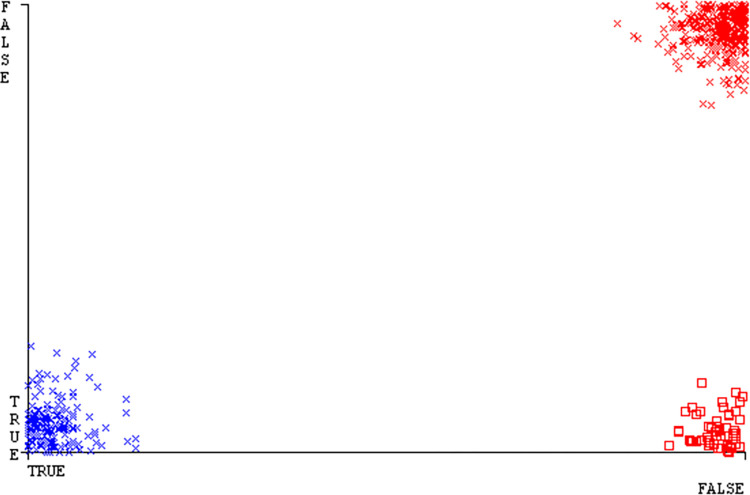
Classifier errors of NBTree based on HDD mono in accuracy & fault prediction.

**Fig 39:**
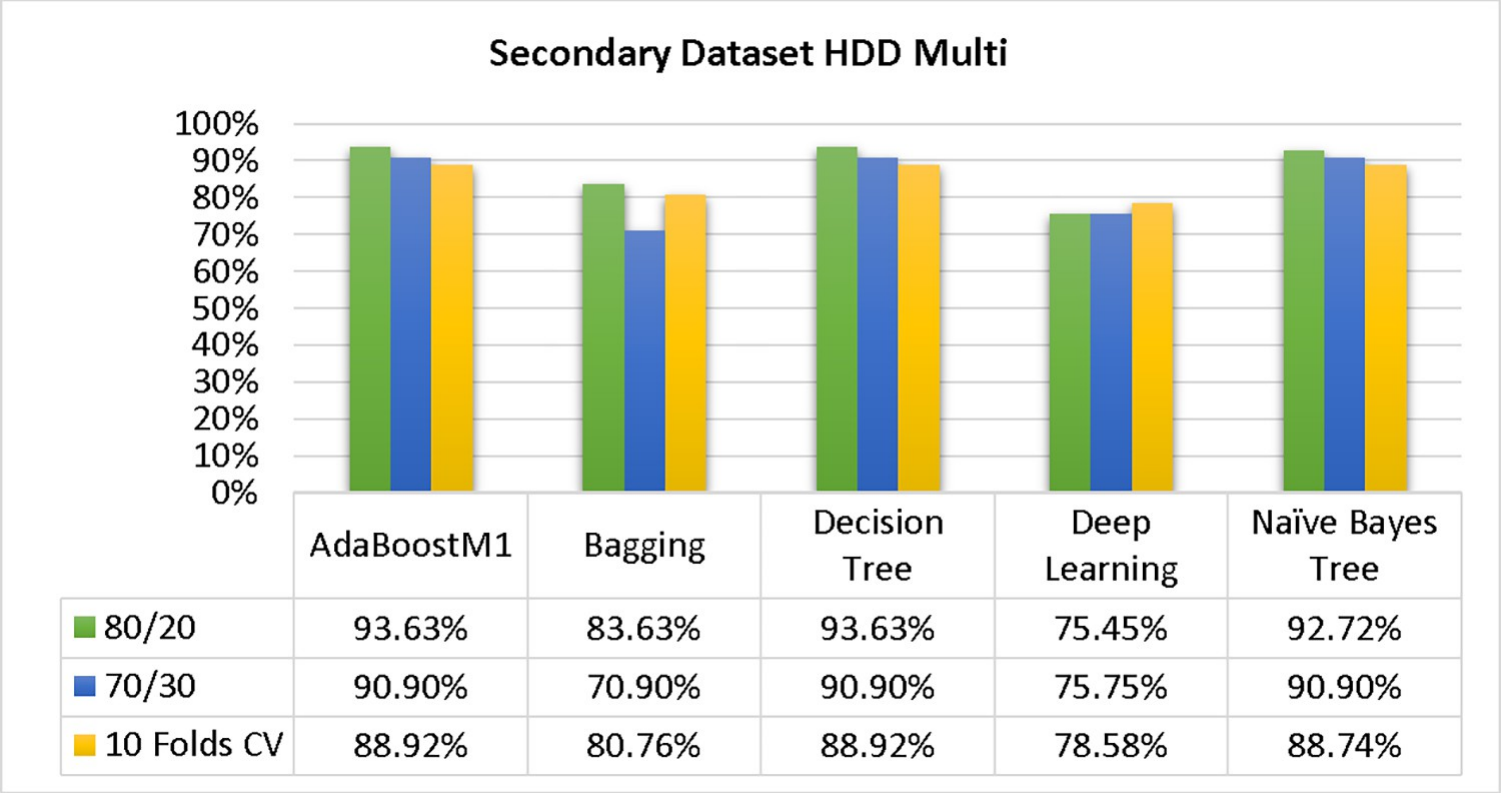
Shows the accuracy of HDD multi on ML classifiers for each class (true/false).

**Fig 40:**
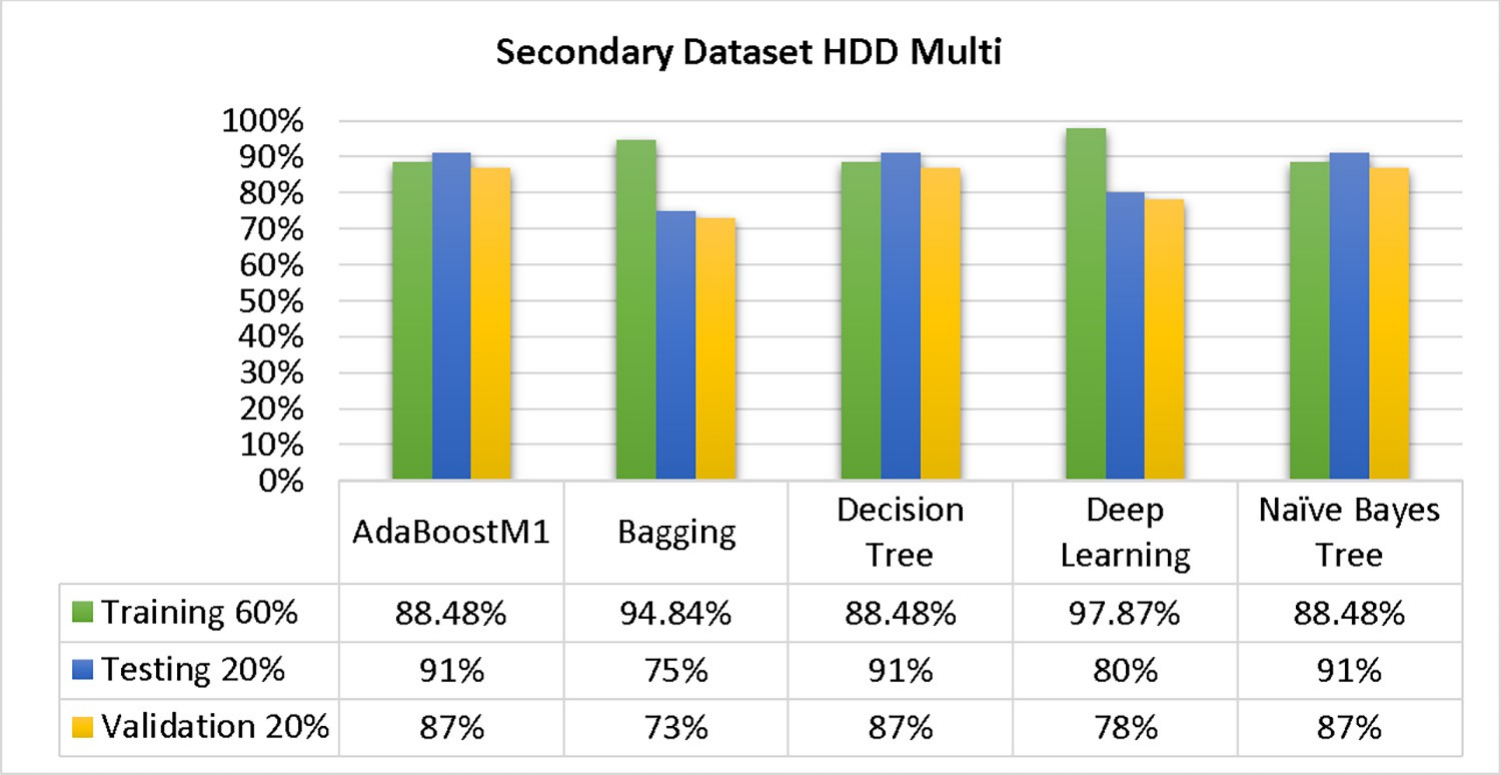
Shows HDD multiclass (true/false) ML classifiers’ accuracy regarding data validation outcomes.

**Fig 41:**
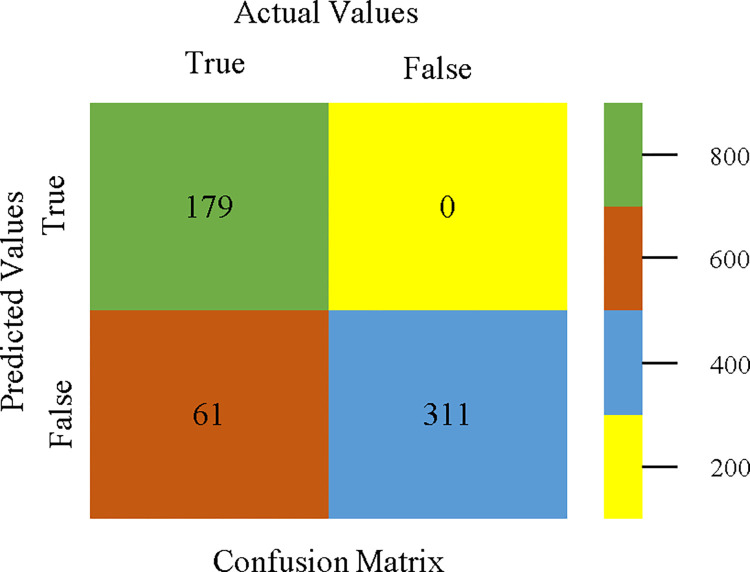
AdaBoostM1 classifier’s confusion matrix for accuracy & fault prediction based on HDD multi.

**Fig 42:**
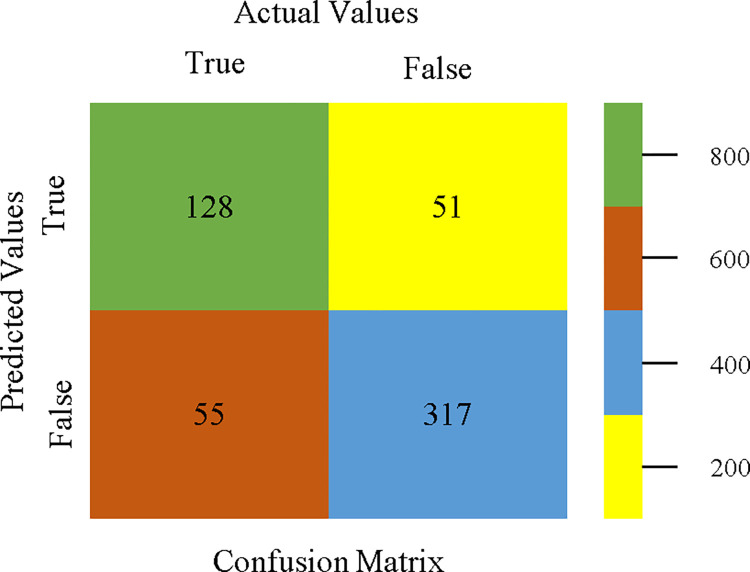
Bagging classifier’s confusion matrix for accuracy & fault prediction based on HDD multi.

**Fig 43:**
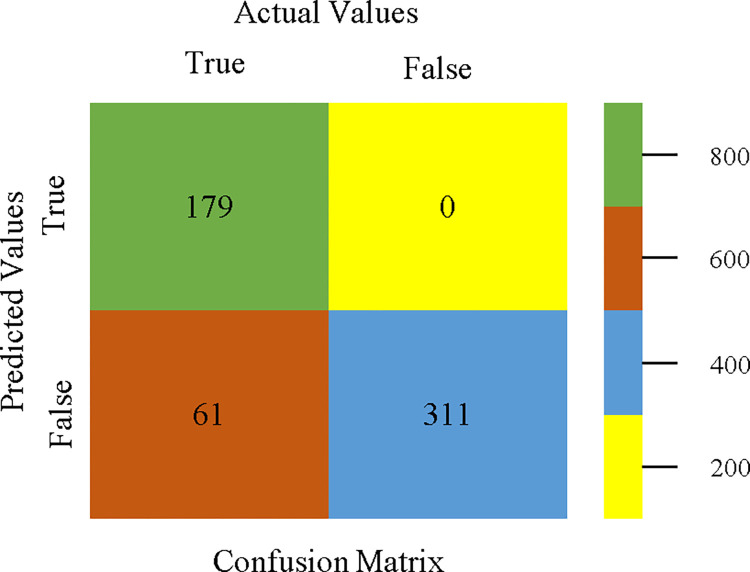
J48 classifier’s confusion matrix for accuracy & fault prediction based on HDD multi.

**Fig 44:**
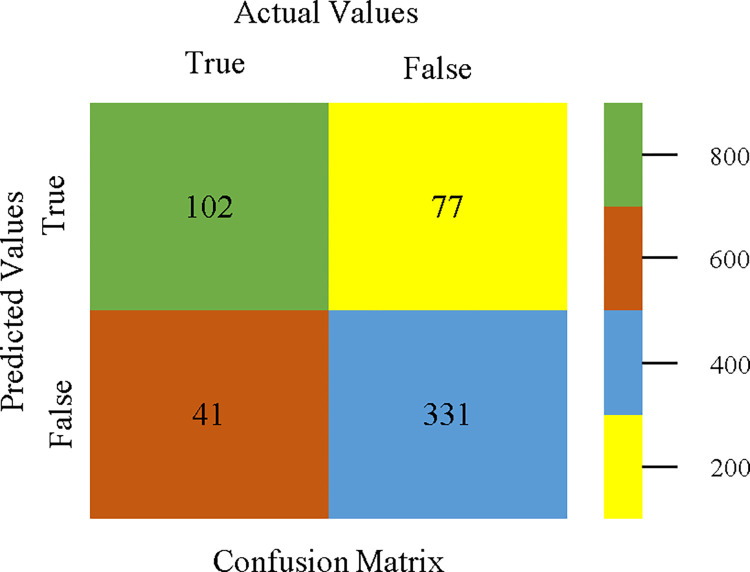
Dl4jMLP classifier’s confusion matrix for accuracy & fault prediction based on HDD multi.

**Fig 45:**
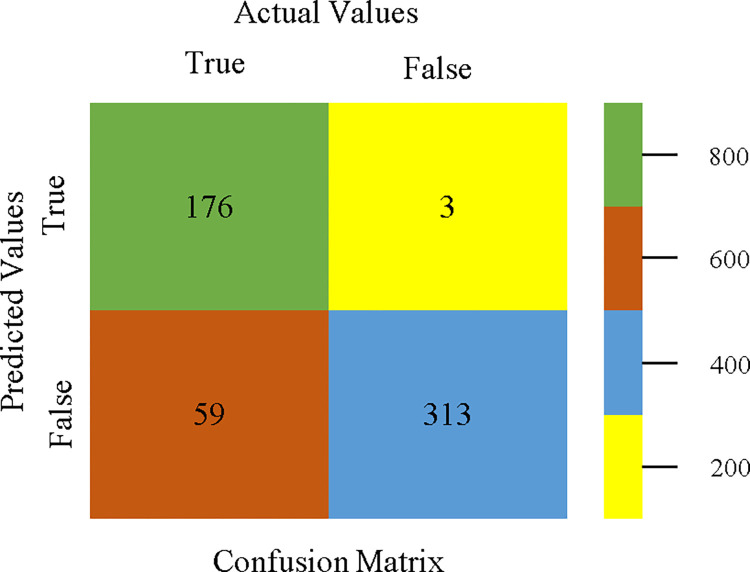
NBTree classifier’s confusion matrix for accuracy & fault prediction based on HDD multi.

**Fig 46:**
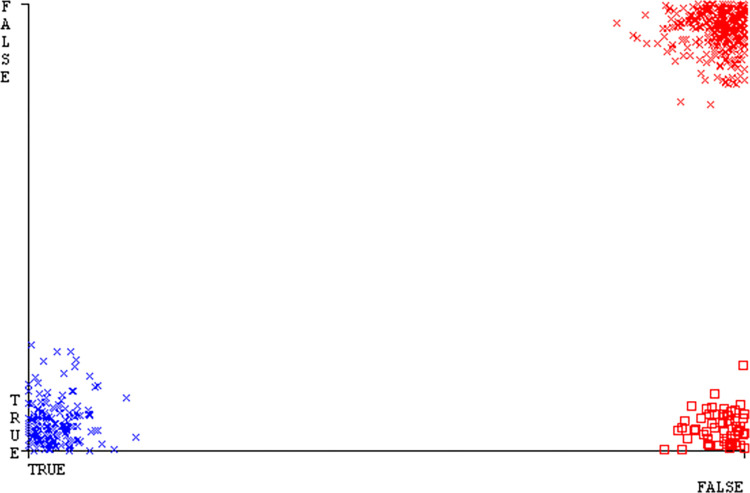
Classifier errors of AdaBoostM1 based on HDD multi-in accuracy & fault prediction.

**Fig 47:**
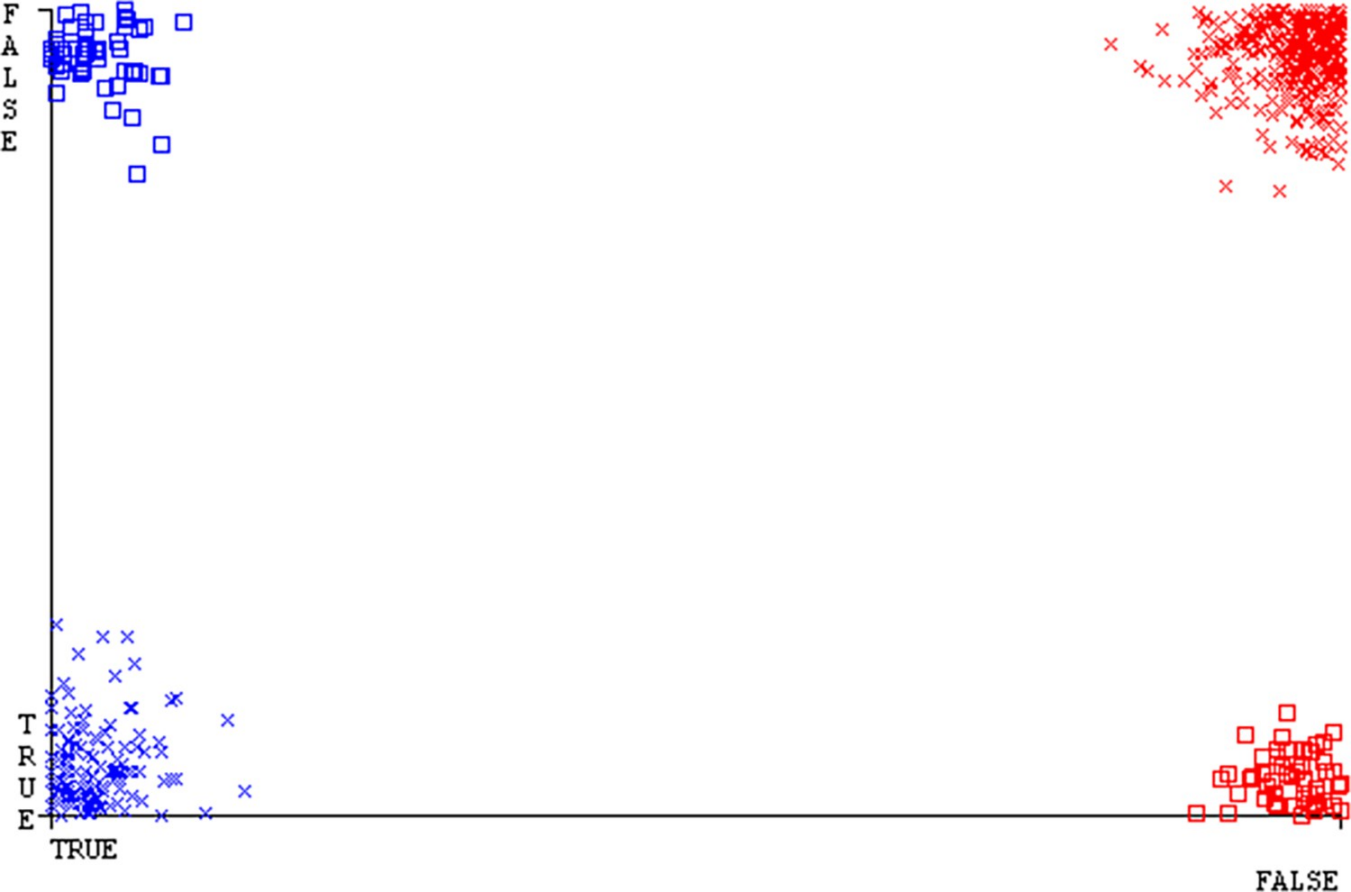
Classifier errors of Bagging based on HDD multi-in accuracy & fault prediction.

**Fig 48:**
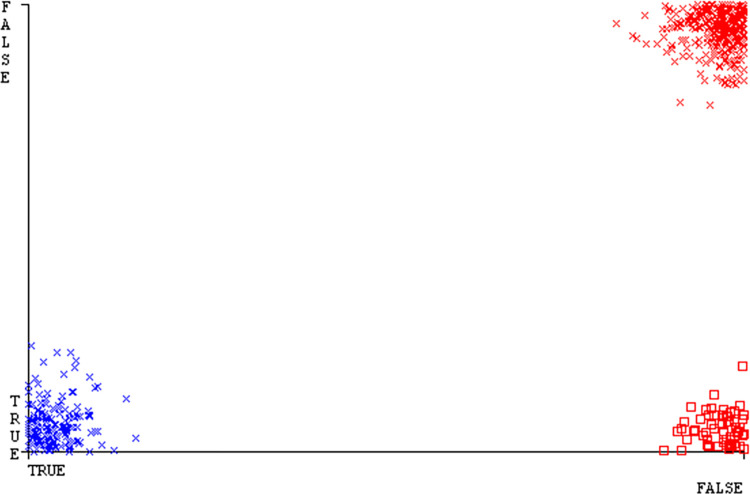
Classifier errors of J48 based on HDD multi-in accuracy & fault prediction.

**Fig 49:**
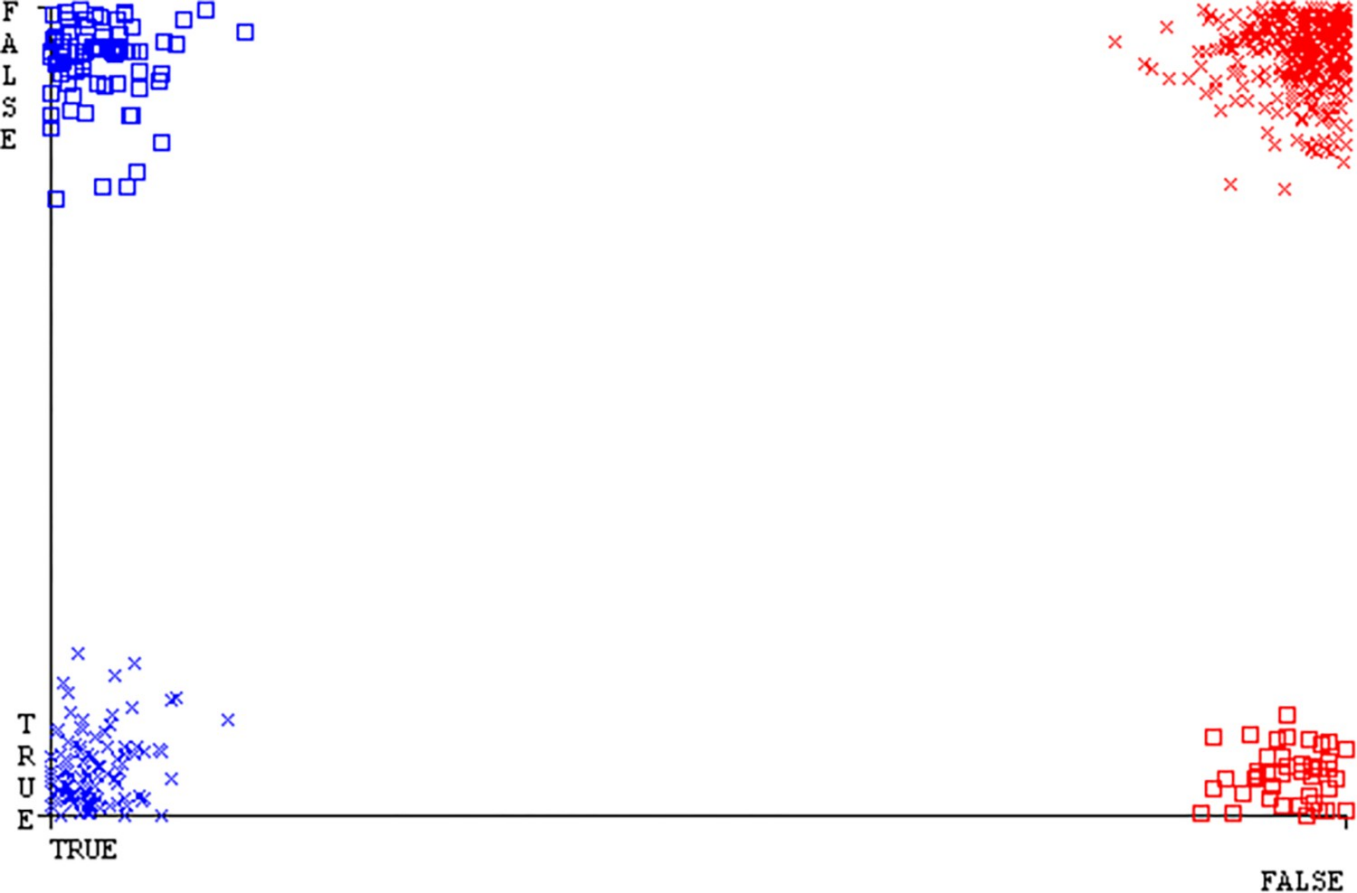
Classifier errors of Dl4jMLP based on HDD multi-in accuracy & fault prediction.

**Fig 50:**
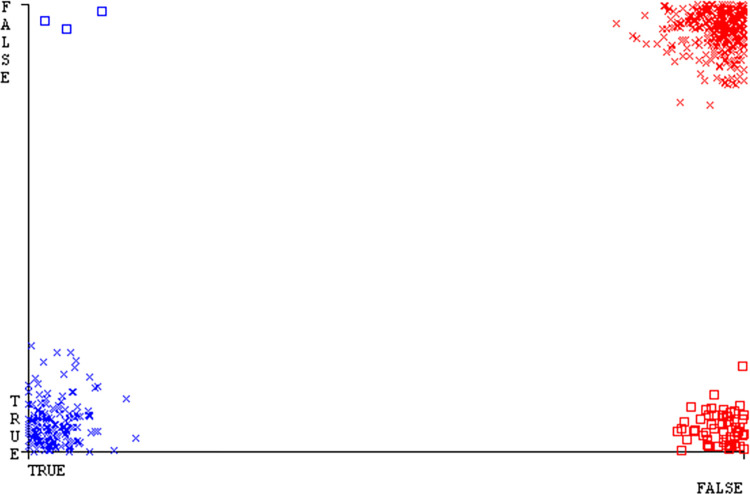
Classifier errors of NBTree based on HDD multi-in accuracy & fault prediction.

### Secondary dataset CPU-mem mono block-I

The study compared the performance of AdaBoostM1, Bagging, J48, Dl4jMLP, & NBTree using Test Split Additional Data Validation & CPU-Mem Mono-related detailed accuracy by class (True/False), as shown in Figs 3 & 4.

The confusion matrix is a useful method for classifying qualities based on qualitative response categories. It is used to compute Accuracy, Precision, Recall, & F-Measure. The confusion matrix for accuracy & fault prediction was obtained using AdaBoostM1, Bagging, J48, Dl4jMLP, & NBTree, & is displayed in Figs 5–9. According to the displayed confusion matrix, the AdaBoostM1 classification model provides the highest accuracy percentage & less fault prediction on CPU-Mem Mono.

The Figs 10–14 show the classifier’s errors, including true positives, true negatives, false positives, & false negatives. The square box indicates the differences between predicted & actual classes.

### Secondary dataset CPU-mem multi block-II

In this study, we compare the detailed accuracy by class (True/False) & prediction on the test split extra data validation of five different models used in CPU-Mem Multi: AdaBoostM1, Bagging, J48, Dl4jMLP, & NBTree. The comparisons are shown in Figs 15 and 16.

The confusion matrix is a useful method for categorizing qualities according to qualitative response categories & is used to compute Accuracy, Precision, Recall, & F-Measure. The confusion matrix for accuracy & fault prediction, obtained using AdaBoostM1, Bagging, J48, Dl4jMLP, & NBTree is displayed in Figs 17–21. According to the confusion matrix that follows, the J48 classification model provides the maximum percentage of accuracy & less fault prediction on CPU-Mem Multi.

The Figs 22–26 display true positive, true negative, false positive, & false negative values for the classifier’s error. The square box shows discrepancies between the actual & anticipated classes.

### Secondary dataset HDD mono block-III

A comparison of the AdaBoostM1, Bagging, J48, Dl4jMLP, & NBTree outcomes in HDD Mono for detailed accuracy by class (True/False) & prediction on test split further data validation is shown in Figs 27 and 28.

The confusion matrix is a helpful approach for categorizing qualities based on qualitative response categories. It is used for computing Accuracy, Precision, Recall, & F-Measure. The confusion matrix for accuracy & fault prediction is achieved by AdaBoostM1, Bagging, J48, Dl4jMLP, & NBTree, & it is displayed in Figs 29–33. According to the corresponding confusion matrix, the J48 classification model provides the maximum percentage of accuracy & minimum defect prediction on HDD Mono.

Figs 34–38 display the classifier’s error, including true positive, true negative, false positive, & false negative values. The square box in the figures illustrates the differences between the actual & anticipated classes.

### Secondary dataset HDD multi block-IV

In this section, we compare the results of AdaBoostM1, Bagging, J48, Dl4jMLP, & NBTree in HDD Multi-related detailed accuracy by class (True/False) & prediction on test split additional data validation. The results are presented in Figs 39 and 40.

To analyze the accuracy, precision, recall, & F-measure, we rely on the confusion matrix. This approach helps categorize qualities based on qualitative response categories. The confusion matrix, obtained using AdaBoostM1, Bagging, J48, Dl4jMLP, & NBTree, for accuracy & fault prediction is displayed in Figs 41–45. According to the confusion matrix, the AdaBoostM1 classification model has the highest percentage of accuracy & the least fault prediction on HDD Multi.

The charts Figs 46–50 illustrate the classifier’s error, including true positive, true negative, false positive, & false negative values. The square box depicts the discrepancies between the actual & predicted classes.

### 2. Models comparison for classification using a primary dataset

Presenting outcomes of classifiers using AdaBoostM1, Bagging, J48, Dl4jMLP, & NBTree based on the STATUS class from the primary dataset.

Based on the primary data findings, the NBTree classifier has the highest accuracy & lower fault prediction percentage among 80/20 (97.05%), 70/30 (96.09%), & 10-fold cross-validation (96.78%) techniques. However, the method complexity of NBTree (1.01 seconds) is not satisfactory. The J48 comes second in terms of accuracy & fault prediction with 80/20 (96.78%), 70/30 (95.95%), & 10-fold cross-validation (96.78%). Moreover, the method complexity of J48 (0.11 seconds) is good. The difference between NBTree & J48 in accuracy & fault prediction is only 0.9%, but there is a 9-second difference in time complexity.

**Fig 51:**
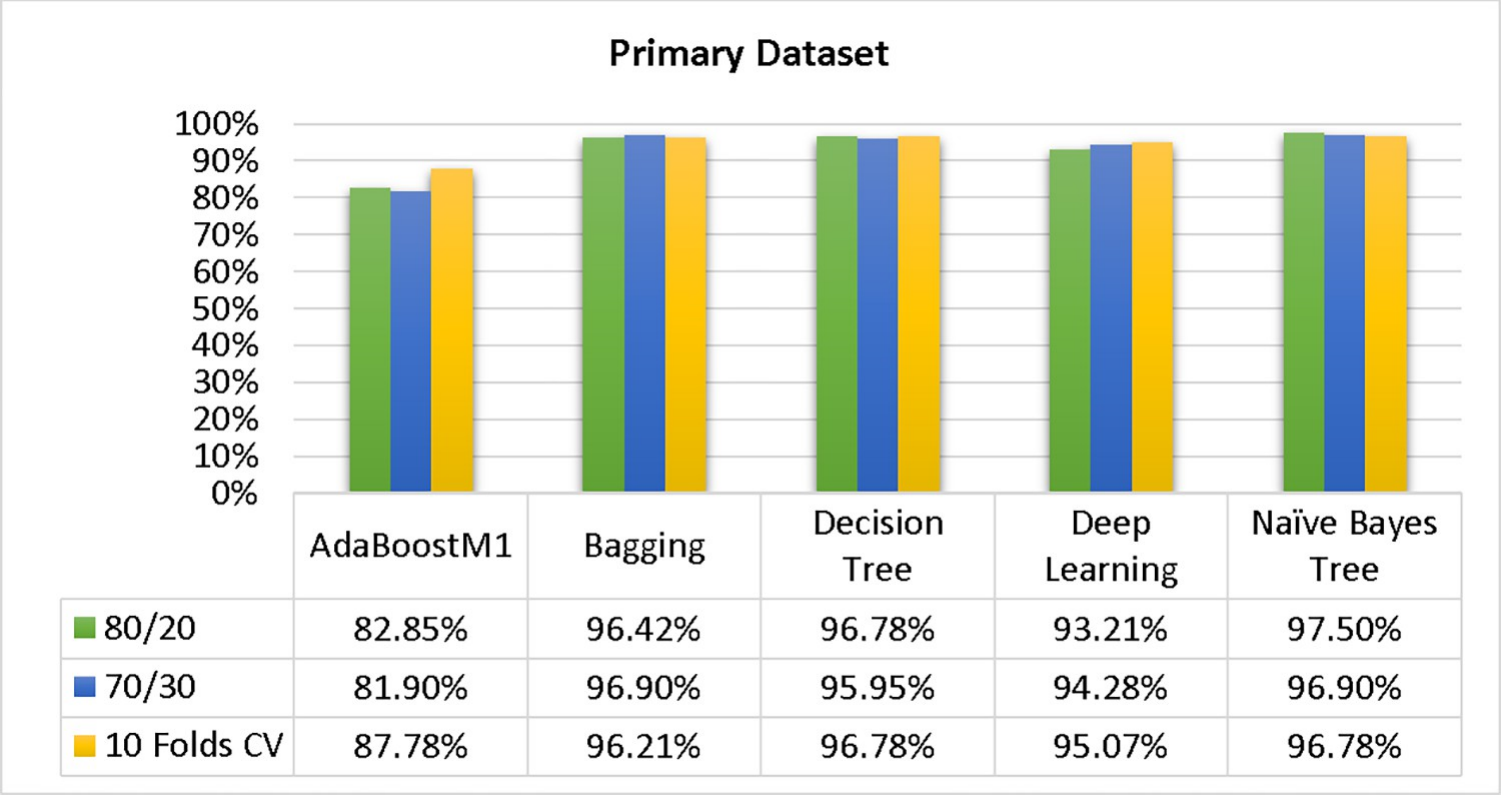
Accuracy of the primary dataset on ML classifiers by class (failure/repair).

**Fig 52:**
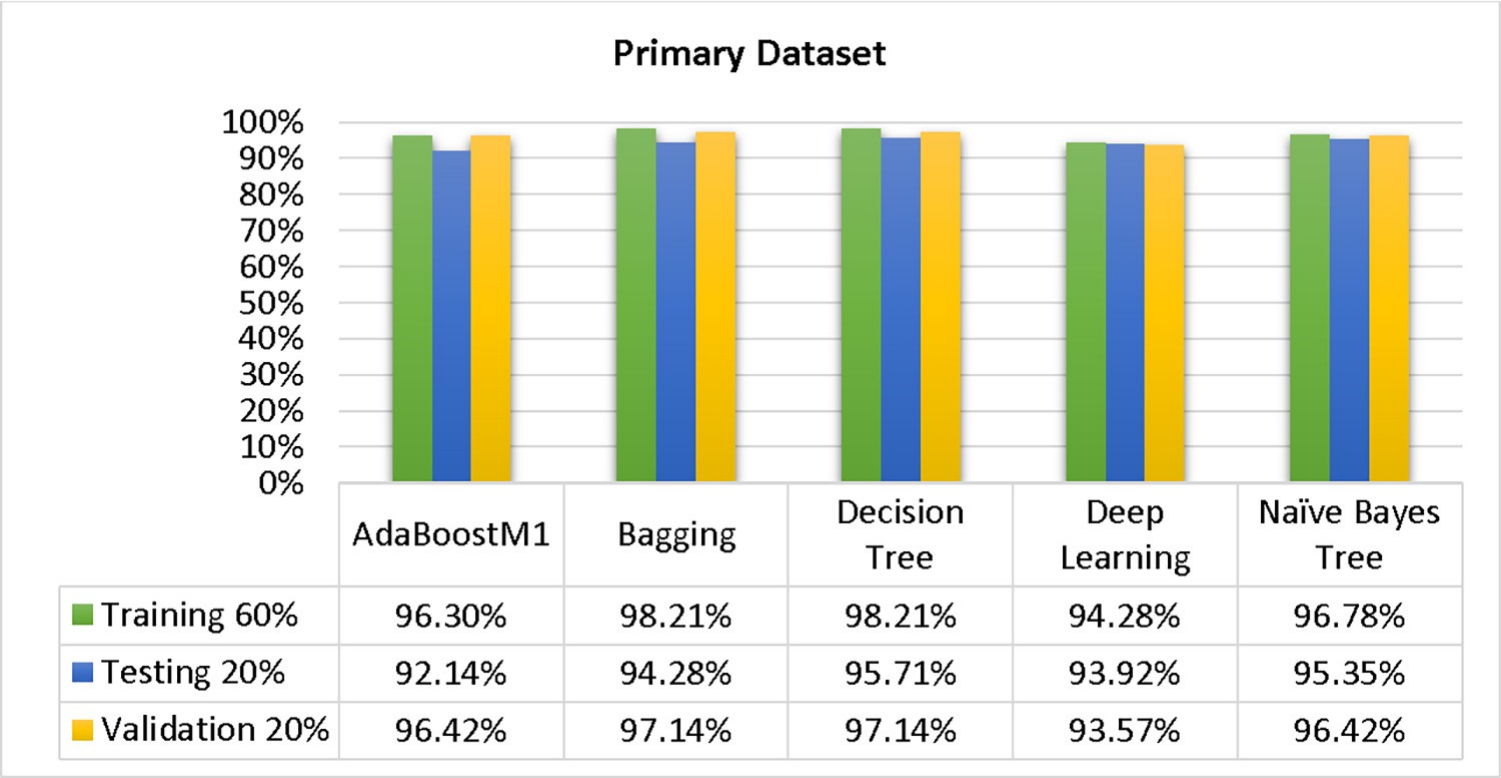
Shows the accuracy by class (failure/repair) of the primary dataset on ML classifiers associated with DV outcomes.

Please find a detailed comparison of the accuracy results for AdaBoostM1, Bagging, J48, Dl4jMLP, & NBTree models. The comparison includes accuracy by class (Repair/Failure) & prediction on the test split. For further data validation, please refer to Figs 51 and 52 of the Primary Dataset.

**Fig 53:**
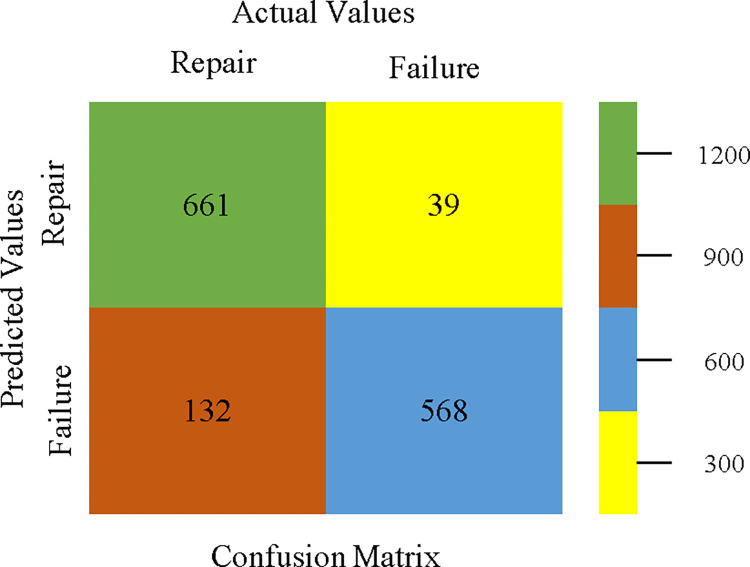
AdaBoostM1 classifier’s confusion matrix for accuracy & fault prediction based on primary dataset.

**Fig 54:**
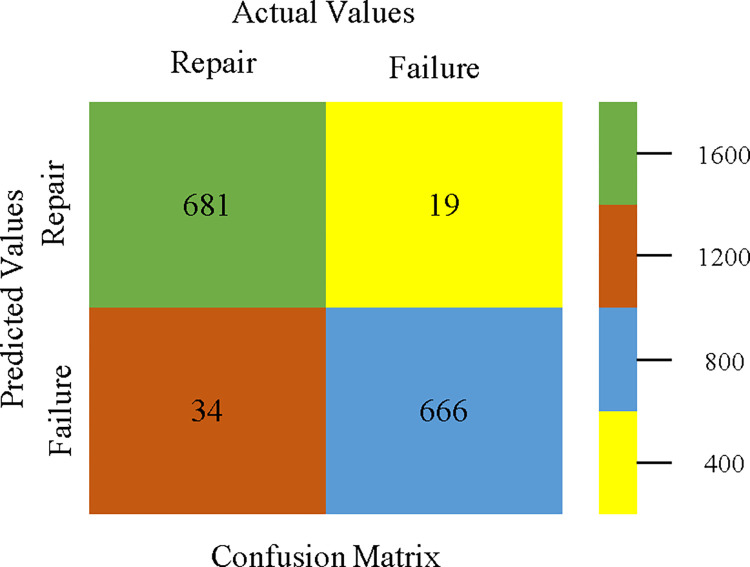
Bagging classifier’s confusion matrix for accuracy & fault prediction based on primary dataset.

**Fig 55:**
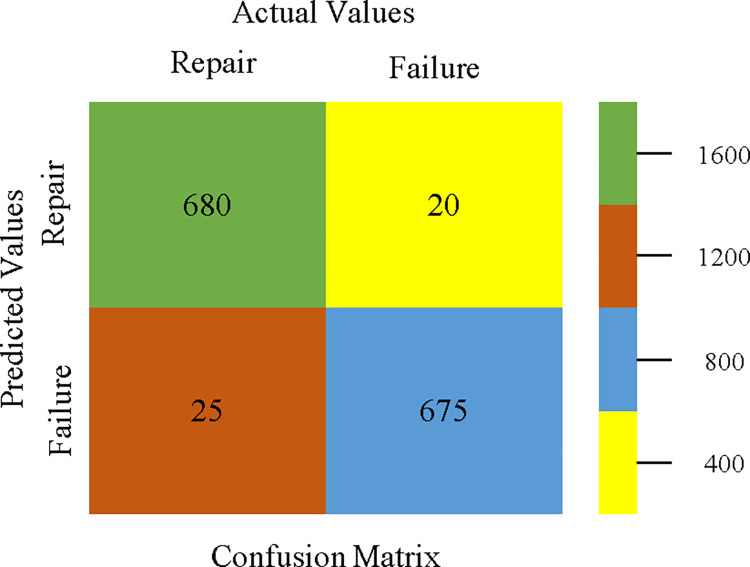
J48 classifier’s confusion matrix for accuracy & fault prediction based on primary dataset.

**Fig 56:**
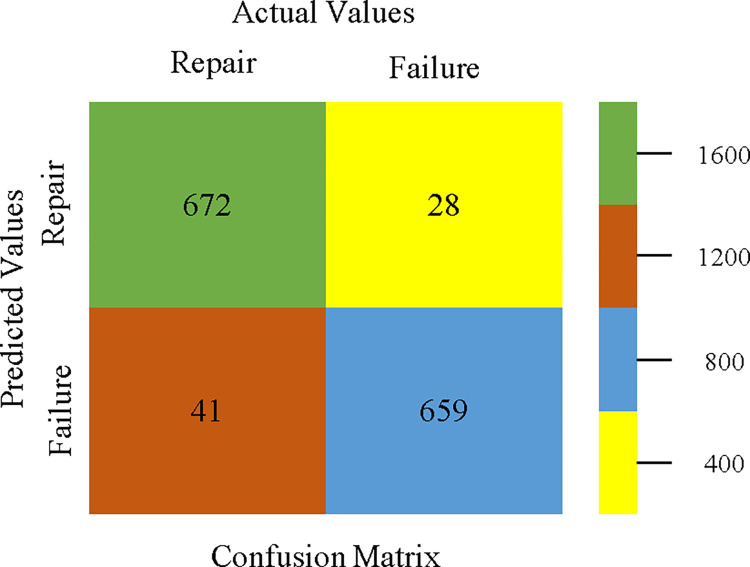
Dl4jMLP classifier’s confusion matrix for accuracy & fault prediction based on primary dataset.

**Fig 57:**
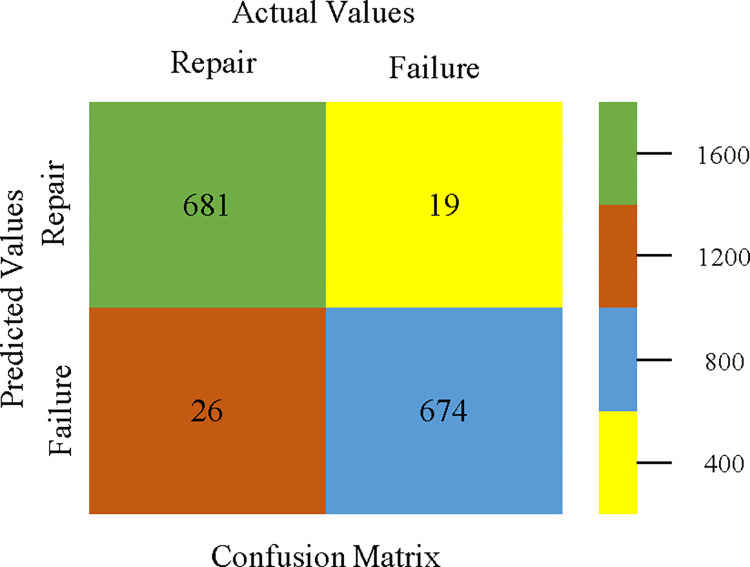
NBTree classifier’s confusion matrix for accuracy & fault prediction based on primary dataset.

The confusion matrix is used to calculate Accuracy, Precision, Recall, & F-Measure. It is used as an efficient technique for the classification of attributes based on qualitative response categories. Figs 53–57 show the confusion matrix related to accuracy & fault prediction, achieved through AdaBoostM1, Bagging, J48, Dl4jMLP, & NBTree. The following confusion matrix indicates that the NBTree classification model gives the highest percentage of accuracy & less fault prediction on the primary dataset, but the algorithm complexity (1.01 seconds) is not good.

**Fig 58:**
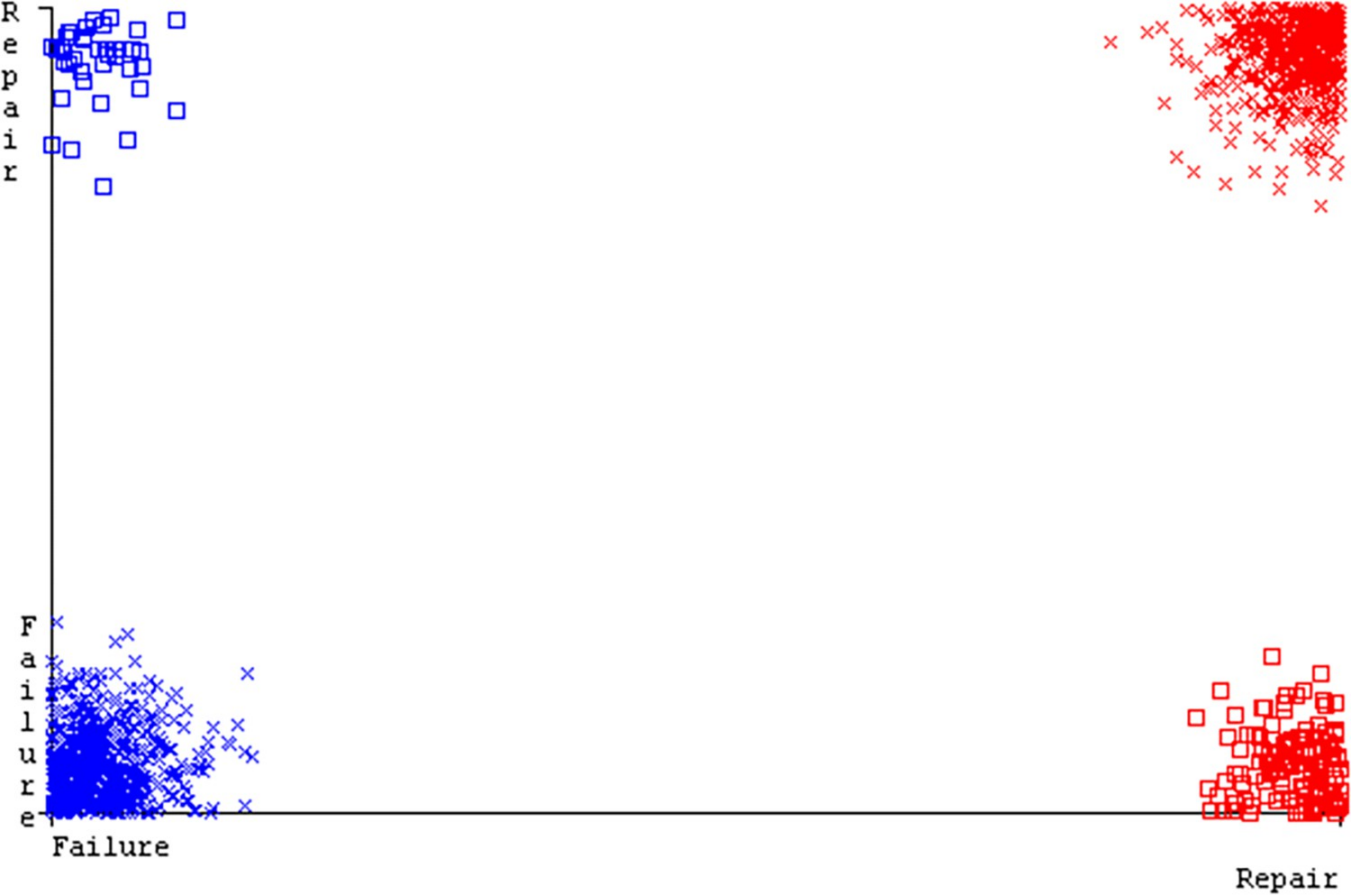
Classifier errors of AdaBoostM1 based on primary data in accuracy & fault prediction.

**Fig 59:**
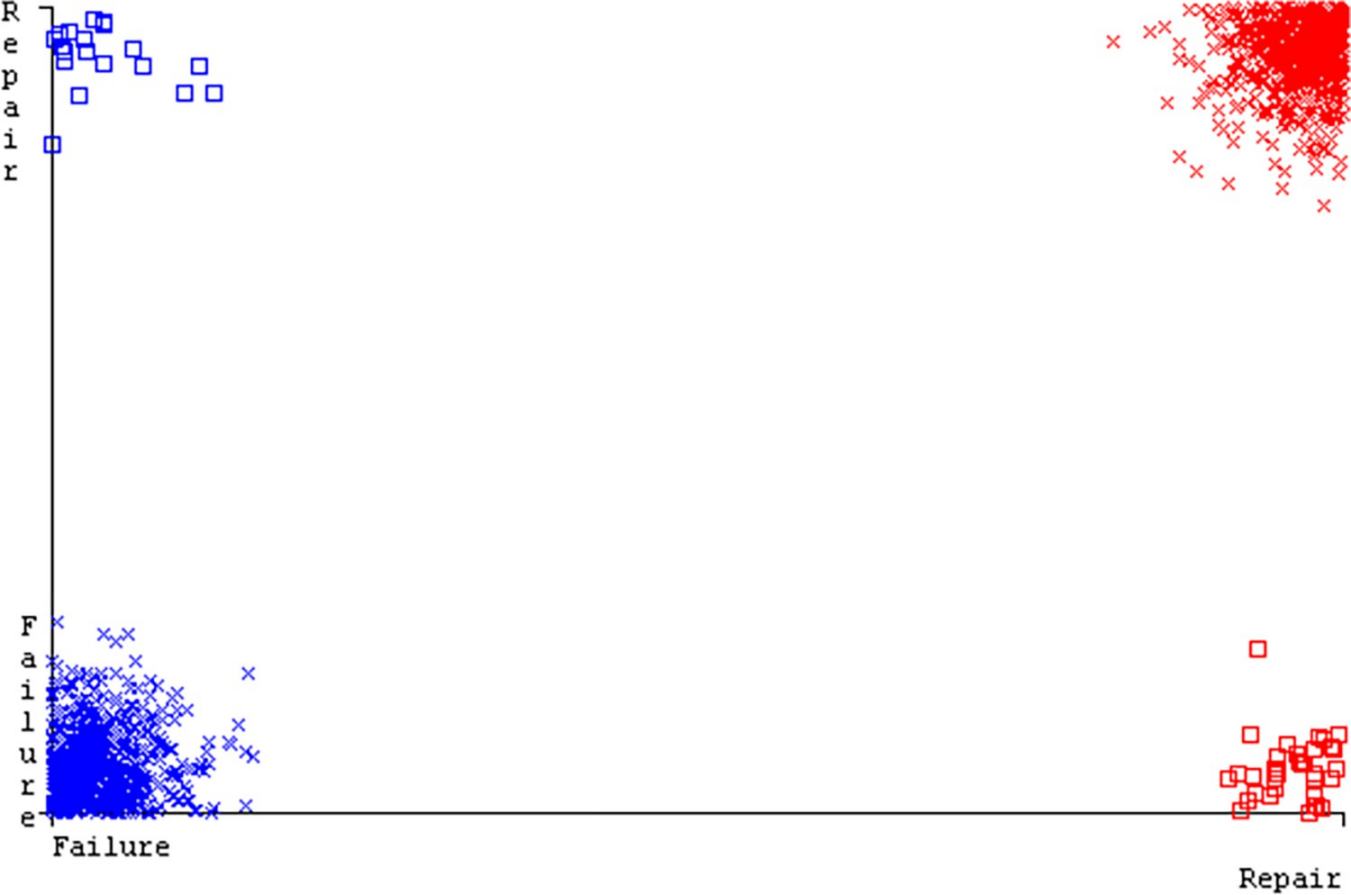
Classifier errors of Bagging based on primary data in accuracy & fault prediction.

**Fig 60:**
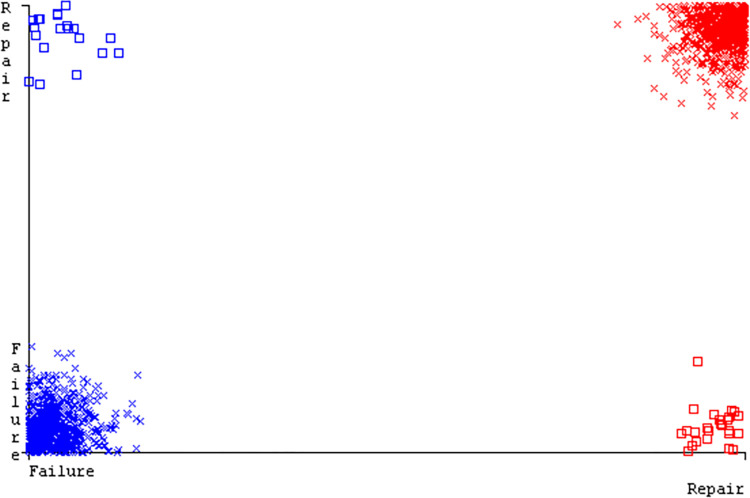
Classifier errors of J48 based on primary data in accuracy & fault prediction.

**Fig 61:**
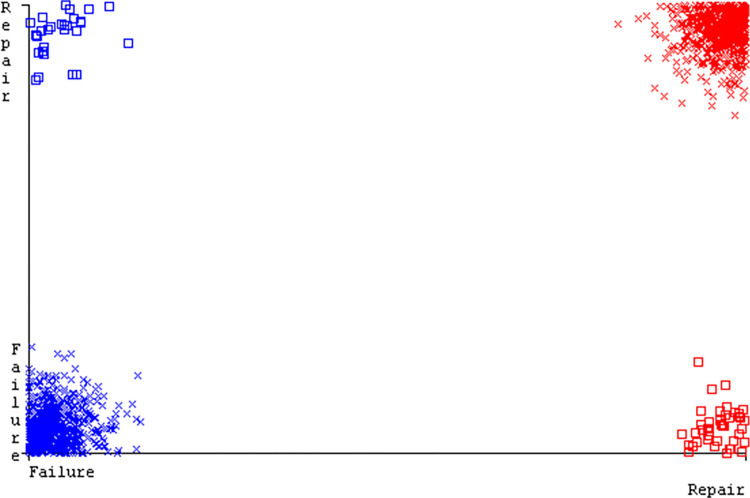
Classifier errors of Dl4jMLP based on primary data in accuracy & fault prediction.

**Fig 62:**
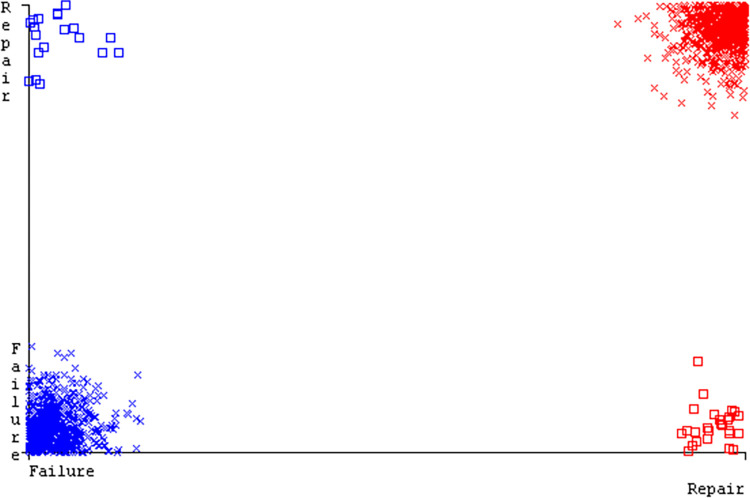
Classifier errors of NBTree based on primary data in accuracy & fault prediction.

The J48 algorithm has the second-highest accuracy & predicts less defects. Its complexity is reasonable, taking only 0.11 seconds. Comparing it with NBTree, the difference in accuracy & fault prediction is just 0.9%. However, the difference in time complexity is significant, taking 9 seconds longer. The classifier’s error is shown by Figs 58–62, which display values for true positive, true negative, false positive, & false negative. The square box in Figs 58–62 shows the discrepancies between the actual & anticipated classes.

### 3. Modified decision tree (J48) results

**Fig 63:**
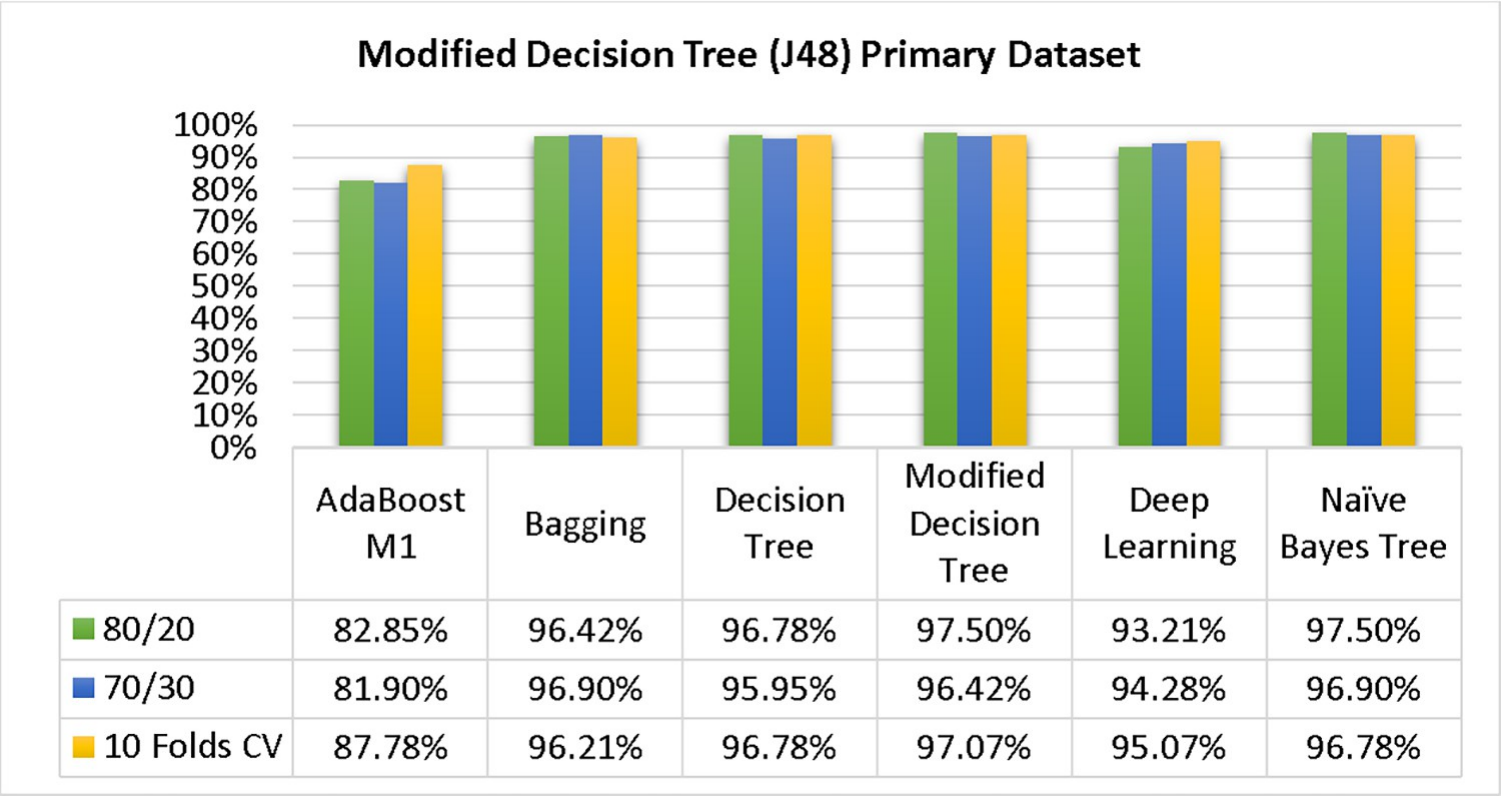
Shows a comparison of ML classifiers with modified decision tree (J48) accuracy based on the primary dataset’s class, (failure/repair).

**Fig 64:**
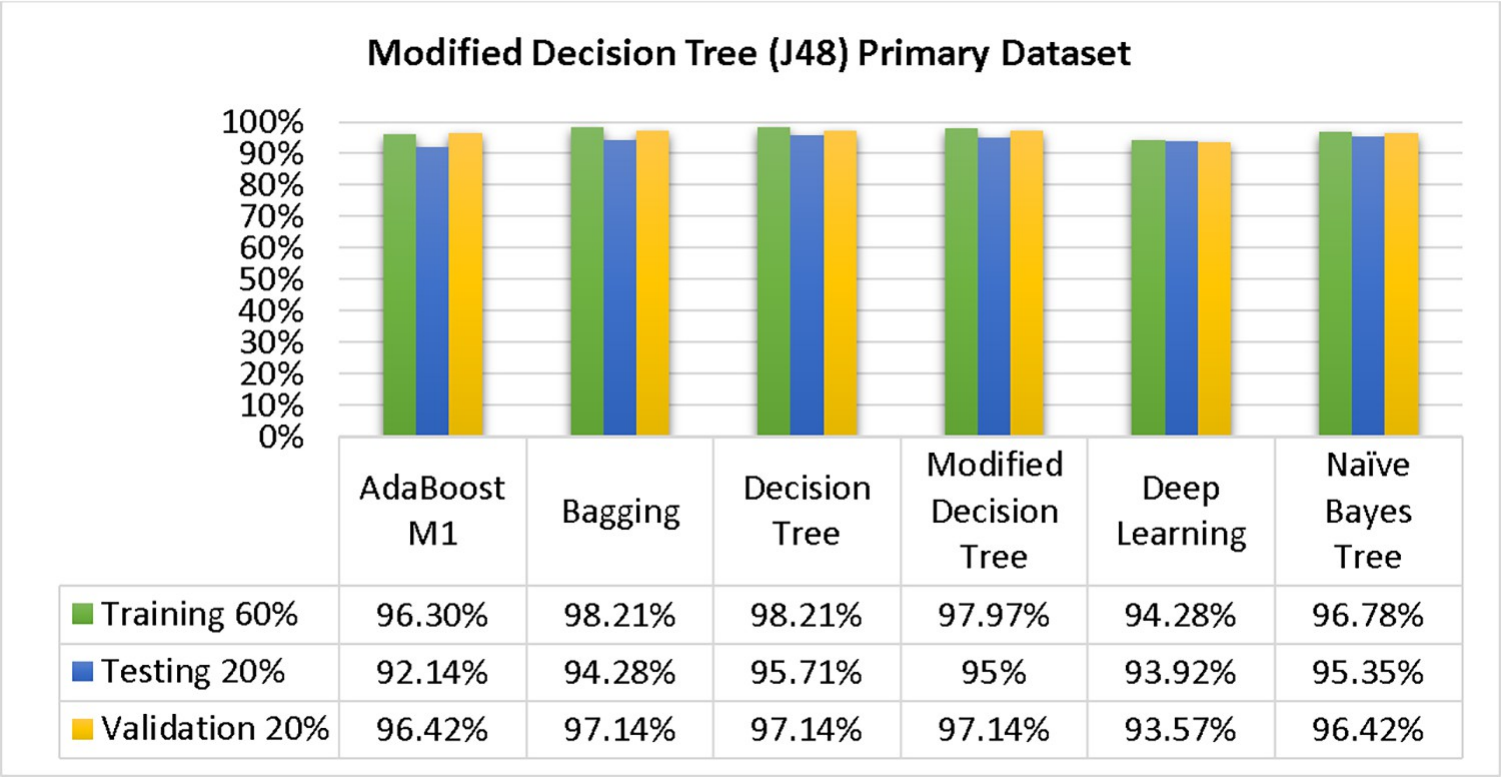
Comparing ML classifiers with modified decision tree (J48) accuracy by primary dataset class about DV findings.

In this subsection, you can see the results of the primary dataset classification in Figs 63 and 64. These results demonstrate that the modified J48 classification model provides the highest accuracy & fewer fault prediction errors when compared to other models. The accuracy of this model is 97.05% for 80/20, 96.42% for 70/30, & 97.07% for 10-fold cross-validation. After the modification, the time complexity of the J48 algorithm has been reduced to 0.02 seconds.

Figs 63 and 64 depicts a comparison of the results of AdaBoostM1, Bagging, J48, Dl4jMLP, & NBTree in the Primary Dataset in terms of detailed accuracy by class (Repair/Failure) & prediction on test split additional data validation.

**Fig 65:**
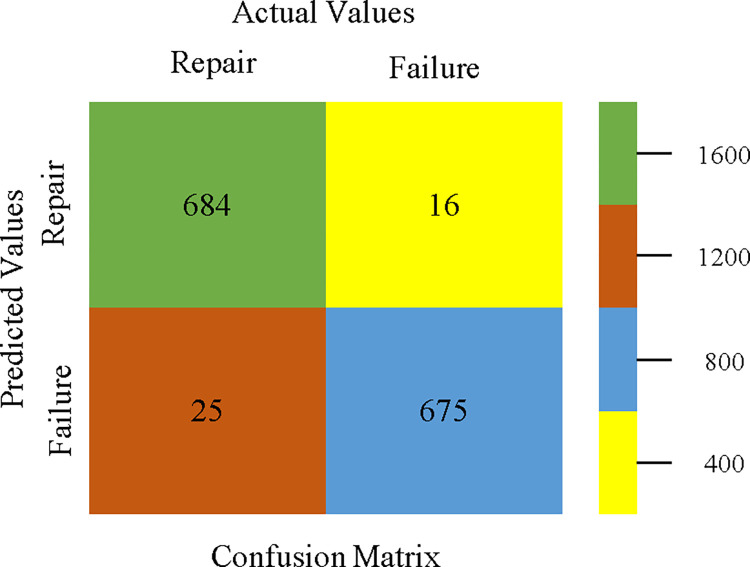
Modified decision tree (J48) classifier’s confusion matrix for accuracy & fault prediction based on primary dataset.

To calculate Accuracy, Precision, Recall, & F-Measure, the confusion matrix is employed. This is a useful technique for classifying qualities based on qualitative response categories. The confusion matrix for accuracy & fault prediction, produced using a modified J48, is shown in Fig 65. According to the confusion matrix, the modified J48 classification model performs better than AdaBoostM1, Bagging, J48, Dl4jMLP, & NBTree in terms of accuracy % & fault prediction error on the primary dataset.

**Fig 66:**
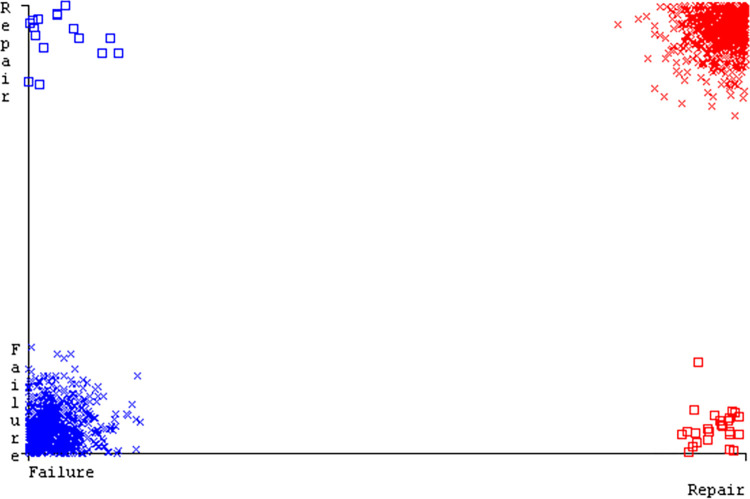
Classifier errors of modified decision tree (J48) based on primary data in accuracy & fault prediction.

Fig 66 shows the classifier’s error, indicating the true positives, true negatives, false positives, & false negatives. It also highlights the differences between the predicted & actual classes.

## Discussion

This study aimed to achieve high accuracy & reliability with minimized error rates. To ensure a smooth implementation of the research, we developed a modified version of the decision tree classifier, J48.

A modified J48 classifier was used to analyze the primary data. The results showed that the suggested approach outperforms the current classifier in terms of accuracy & fault prediction. The acquired results were compared with those of the current AdaBoostM1, Bagging, J48, Dl4jMLP, & NBTree classifiers. To evaluate the classifier’s performance, high accuracy with low fault prediction is considered the most crucial criterion.

The proposed classifier was compared to AdaBoostM1, Bagging, J48, Dl4jMLP, & NBTree classifiers, & it was found that the proposed classifier outperformed the existing classifiers in terms of accuracy & speed. The proposed classifier was successful in classifying 97.07% of instances correctly. The novelty of the proposed research lies in its unique set of methods that have been associated with reduced fault prediction & high accuracy to ensure dependability. A modified J48 was suggested using parameter tweaking, which is considered to be a distinct strategy.

Using the WEKA tool, the experiment is being simulated. A group of machine learning algorithms is known as WEKA. WEKA provides tools for preprocessing, classifying, regressing, clustering, generating association rules, and visualizing data. Weka is distributed under the General Public License (GNU), making it open-source software. It works well for creating new machine-learning systems. The algorithms can be invoked from your own Java code or applied directly to a dataset [31].

## Conclusions and future work

The study’s findings were associated with several classifiers that accurately identified errors using "STATUS" in primary data and "ISFAULT" in secondary data.

In the study, the AdaBoostM1 classifier found that the secondary data findings (CPU-Mem Mono) had the highest accuracy rate with the fewest fault predictions. The accuracy rate for CPU-Mem Mono was 77.87% for 80/20, 77.01% for 70/30, and 77.06% for 10-fold cross-validation. On the other hand, the J48 classifier indicated that the secondary data findings (CPU-Mem Multi) had the best accuracy rate while predicting fewer faults. CPU-Mem Multi’s accuracy rate was 89.71% for 80/20, 90.28% for 70/30, and 92.82% for 10-fold cross-validation. It was observed that the AdaBoostM1 classifier had the highest accuracy and the lowest fault prediction among the HDD multi-classifiers. The accuracy rates were as follows: 93.63% for 80/20, 90.09% for 70/30, and 88.92% for 10-fold cross-validation. The J48 classifier performed the best, achieving the highest accuracy and lowest fault prediction rates for (HDD Mono) at 80/20 (90.35%), 70/30 (92.35%), and 10-fold cross-validation (90.49%).

The NBTree classifier has the lowest fault prediction rate and the highest accuracy percentages in the primary data findings (80/20–97.05%, 70/30–96.09%, and 10-fold cross-validation—96.78%). However, its technique complexity is modest, taking 1.01 seconds to execute. On the other hand, J48 has the second-highest accuracy in terms of 80/20 (96.78%), 70/30 (94.95%), and 10-fold cross-validation (96.78%). It also has the least amount of fault prediction and a decent technique complexity of 0.9 seconds. The difference between NBTree and J48 is only 0.9% in terms of accuracy and fault prediction, and 0.9 seconds in time complexity.

### Accomplishment of the objectives

With the assistance of the literature review & research objectives presented in Table 6, we have achieved high accuracy & less errors in predicting faults in CC.

**Table 6:** Achievement of research aims.

S.No	Objective	Input & Output	Achievements
1	Find the best ML classifiers to improve the accuracy of predicting failures.	We have conducted a comprehensive review of existing literature. To search for articles, we used the following keywords in databases like Google Scholar, Web of Science, & Science Direct: popular ML techniques that can help achieve high accuracy & reduce fault prediction errors, ML classifiers, & ML approaches for fault classification & prediction in CC, among others.	After conducting a thorough analysis, we have identified the most effective ML classifiers that result in reduced fault prediction errors & high accuracy.
2	To mitigate low accuracy & high errors in failure prediction, an ML algorithm is recommended.	To achieve optimal results, select a precise ML classifier that delivers high accuracy rates with minimal error predictions & then make any necessary adjustments.	After identifying the J48 classifier that provided high accuracy & fewer fault prediction errors, we modified it to achieve optimal results.

### Contribution to cloud computing

A recent update has been made to the J48 classifier. This update is particularly beneficial for CC applications, as it significantly improves accuracy & reduces the number of failure prediction errors for consumers. Achieving this high level of accuracy & fault prediction reliability was a challenging task. However, we were able to accomplish this by adjusting the confidence factor parameter & do not making split point actual value, which resulted in improved accuracy, mean square error, & fitness.

This study demonstrates how ML can improve CC by reducing prediction errors & achieving high accuracy for consumers.

### Restrictions

1. As this is an HPC fault dataset, we can collect Antarex secondary data, however, it will require additional processing power. Alternatively, we can obtain this dataset through the ZONODO website.
2. To produce a fault dataset for primary data production, the Weibull distribution was not utilized.
3. An attempt was made to obtain the primary dataset using the Weibull distribution.

### Future directions

1. The CloudSim primary dataset can be generated through a graphical user interface that uses the Weibull distribution technique.
2. Code can be automatically modified to tune parameters, but should not be stopped to discover optimal values.
3. Using NBTree can achieve high accuracy & low fault prediction errors, but further study on the algorithm’s complexity is necessary. This study can also be used for comparative analysis.
4. Deep learning algorithms can be used to predict fewer errors with high accuracy. However, to achieve more accurate & reliable results, a larger sample size is required. Deep learning techniques outperform ML methods when the dataset is huge.
